# SALL1 Modulates CBX4 Stability, Nuclear Bodies, and Regulation of Target Genes

**DOI:** 10.3389/fcell.2021.715868

**Published:** 2021-09-21

**Authors:** Immacolata Giordano, Lucia Pirone, Veronica Muratore, Eukene Landaluze, Coralia Pérez, Valerie Lang, Elisa Garde-Lapido, Monika Gonzalez-Lopez, Orhi Barroso-Gomila, Alfred C. O. Vertegaal, Ana M. Aransay, Jose Antonio Rodriguez, Manuel S. Rodriguez, James D. Sutherland, Rosa Barrio

**Affiliations:** ^1^Center for Cooperative Research in Biosciences (CIC bioGUNE), Basque Research and Technology Alliance, Derio, Spain; ^2^Viralgen Vector Core, Parque Científico y Tecnológico de Guipúzcoa, San Sebastián, Spain; ^3^Department of Cell and Chemical Biology, Leiden University Medical Center, Leiden, Netherlands; ^4^Centro de Investigación Biomédica en Red. Enfermedades Hepáticas y Digestivas (CIBERehd), Instituto de Salud Carlos III, Madrid, Spain; ^5^Department of Genetics, Physical Anthropology and Animal Physiology, University of the Basque Country, Leioa, Spain; ^6^Laboratoire de Chimie de Coordination-CNRS, Paul Sabatier: Université Toulouse III, Toulouse, France

**Keywords:** CBX4, SALL1, nuclear bodies, SUMO, ubiquitin

## Abstract

Development is orchestrated through a complex interplay of multiple transcription factors. The comprehension of this interplay will help us to understand developmental processes. Here we analyze the relationship between two key transcription factors: CBX4, a member of the Polycomb Repressive Complex 1 (PRC1), and SALL1, a member of the Spalt-like family with important roles in embryogenesis and limb development. Both proteins localize to nuclear bodies and are modified by the small ubiquitin-like modifier (SUMO). Our results show that CBX4 and SALL1 interact in the nucleoplasm and that increased SALL1 expression reduces ubiquitination of CBX4, enhancing its stability. This is accompanied by an increase in the number and size of CBX4-containing Polycomb bodies, and by a greater repression of CBX4 target genes. Thus, our findings uncover a new way of SALL1-mediated regulation of Polycomb bodies through modulation of CBX4 stability, with consequences in the regulation of its target genes, which could have an impact in cell differentiation and development.

## Introduction

Development of higher organisms is orchestrated by a complex interplay of regulatory networks involving multiple signaling pathways and transcriptional regulatory factors. Two key families of transcriptional repressor proteins involved in development are the Polycomb Group (PcG) and the Spalt-like (SALL) proteins.

Polycomb Group proteins are involved in epigenetic regulation and control cell fate during embryonic development. These proteins accumulate in nuclear foci called Polycomb (Pc) bodies, which are involved in transcriptional repression (Saurin et al., 1998; Cheutin and Cavalli, 2012; Entrevan et al., 2016; Schuettengruber et al., 2017) and form two distinct complexes: Polycomb Repressive Complex 1 and 2 (PRC1 and PRC2), conserved from flies to human. A crucial component of the PRC1 complex is CBX4. CBX4 is required to maintain the transcriptionally repressive state of HOX genes during development, and has an important role in several essential pathways. Thus, it has been described to facilitate differentiation of hematopoietic stem cells (Klauke et al., 2013), counteracting cellular senescence (Ren et al., 2019) and maintaining the epithelial lineage identity *via* repression of non-epidermal lineage and cell cycle inhibitor genes (Mardaryev et al., 2016). Moreover, CBX4 is recruited rapidly to sites of DNA damage (Ismail et al., 2012) and has emerged as a critical component of the DNA end resection machinery (Soria-Bretones et al., 2017).

Spalt-like family members (SALL1 to SALL4), on the other hand, are important regulators of animal development, being crucial for the formation of the limbs, kidneys, and the central and peripheral nervous systems, among other organs (de Celis and Barrio, 2009). SALL proteins are characterized by the presence of several precisely spaced copies of the zinc finger domain (de Celis and Barrio, 2009). They also contain a N-terminal glutamine-rich region, which could have a role in dimerization or protein–protein interactions (Kohlhase et al., 1998; Buck et al., 2000; Sweetman et al., 2003; Borozdin et al., 2006), and a conserved N-terminal motif that mediates its interaction with one of the major corepressor complexes in mammalian cells, the nucleosome remodeling deacetylase (NuRD) complex (Kiefer et al., 2002; Lauberth and Rauchman, 2006). Like the PcG proteins, SALL1 and its homologs localize in nuclear bodies, as it has been reported in cultured cells and *in vivo* (Netzer et al., 2001; Kiefer et al., 2002; Sánchez et al., 2010; Abedin et al., 2011). However, the nature and function of these bodies have not been explored.

CBX4 and SALL1 play important roles in different aspects of human health. Dysregulation of CBX4 contributes to the occurrence and progression of human tumors, in which it can act as either oncogene or tumor suppressor, depending on the cellular context (Wang et al., 2016). Mutations in *SALL1*, on the other hand cause Townes–Brocks Syndrome (TBS), an autosomal dominant syndrome characterized by renal anomalies, hearing loss, congenital heart defects, and eye anomalies among other symptoms (Kohlhase, 1993). TBS-causing mutations produce truncated SALL1 proteins lacking most of the zinc finger pairs, which aberrantly localize to the cytoplasm and interfere with centrosomal components, resulting in the formation of longer and more abundant primary cilia in patient-derived cells (Bozal-Basterra et al., 2018, 2020).

As described for many other transcriptional regulatory factors, the localization and activity of CBX4 and SALL1 can be modulated by post-translational modifications, including conjugation to ubiquitin or ubiquitin-like (UbL) proteins, such as small ubiquitin-like modifier (SUMO). Thus, CBX4 is SUMOylated and it is a substrate of the SUMO-deconjugating enzyme SENP2 (Wotton and Merrill, 2007; Kang et al., 2010). In addition, it was identified as a SUMO substrate in different proteomic analyses (Golebiowski et al., 2009; Galisson et al., 2011; Hendriks et al., 2014, 2015; Lamoliatte et al., 2014; Tammsalu et al., 2014; Xiao et al., 2015; Hendriks and Vertegaal, 2016). Interestingly, CBX4 itself is proposed to be a SUMO E3 ligase, and is involved in SUMOylation of the transcriptional corepressor C-terminal-binding protein (CtBP) (Kagey et al., 2003), the nucleocytoplasmic shuttling protein hnRNP (Pelisch et al., 2012), the transcriptional co-activator Prdm16 (Chen et al., 2018), and other chromatin-associated factors including CTCF, Dnmt3a, or Bm1 (Li et al., 2007; MacPherson et al., 2009; Ismail et al., 2012). CBX4 has also been found ubiquitinated and its polyubiquitination influences the dynamics of the PRC1 at the chromatin and the regulation of downstream genes (Povlsen et al., 2012; Mertins et al., 2013; Udeshi et al., 2013; Ning et al., 2017; Akimov et al., 2018; Wang et al., 2020).

In the case of SALL1, interaction with SUMO1 and the SUMO E2 conjugase UBC9 has been reported using yeast two-hybrid and *in vitro* assays, with SUMOylation mapped to lysine 1086 (Netzer et al., 2002). Subsequently, SALL1 as well as other SALL proteins, have been confirmed as targets of SUMOylation by proteomics analyses (Golebiowski et al., 2009; Galisson et al., 2011; Hendriks et al., 2014, 2015; Schimmel et al., 2014; Xiao et al., 2015; Hendriks and Vertegaal, 2016). In *Drosophila*, SUMOylation of SALL homologs influences their role in vein pattern formation in the wing and their transcriptional repressor activity (Sánchez et al., 2010, 2011).

Remarkably, although different functional aspects of CBX4 and SALL1 have been addressed in previous studies, a regulatory interplay between these proteins has not been described so far. Interestingly, we identified CBX4, as well as other PcG proteins, as a possible interactor of SALL1 by proximity proteomics (Bozal-Basterra et al., 2018). In addition, *sall* genes and *Pc* interact genetically in *Drosophila*, as mutations in the homolog *spalt-major* enhanced the phenotypical effects of Pc group mutations during embryogenesis (Casanova, 1989; Landecker et al., 1994). These findings, together with the localization of both proteins to nuclear bodies, as well as the regulation by SUMO of both proteins, prompted us to further investigate a potential functional or regulatory interplay between SALL1 and CBX4. We report here a novel interaction between these two transcriptional regulators in the nucleoplasm. Interestingly, SALL1 influences the stability of CBX4 by modulating its ubiquitination, which might be related to changes in the regulatory capacity of CBX4 over HOX genes. Overall, we present here a novel mechanism of regulation of a crucial factor in development, which has consequences for the regulation of its target genes.

## Materials and Methods

### Cell Culture and Cell Transfection

Human U2OS (ATCC HTB-96) and HEK 293FT (Invitrogen) cells, as well as derived cell lines, were cultured at 37°C with 5% of CO2 in DMEM (Dulbecco’s modified Eagle’s medium; Gibco) supplemented with 10% FBS and 1% penicillin/streptomycin (Gibco). HEK 293FT cells were transiently transfected using calcium phosphate in 10 cm dishes with 3–10 μg of DNA using different sets of plasmids according to each experiment. Briefly, DNA was mixed with 500 μl of 2.5 M CaCl_2_ and H_2_O (1:10). The was added drop by drop to the same volume of HBS (NaCl 280 mM, KCl 10 mM, Na2HPO4 1.5 mM, glucose 12 mM, HEPES 50 mM), incubated for 10–15 min, and added to the cells. U2OS cells were transiently transfected using PEI (Sigma Aldrich #408727), or Effectene (Qiagen) according to the manufacturers’ instructions.

### Generation of Plasmids

The following plasmids were used in this study (Table 1). DNA fragments were amplified from the indicated plasmids by high-fidelity PCR Platinum SuperFi (Thermo). PCR products were purified using mi-Gel Extraction kit (Metabion), digested if necessary using the restriction enzymes (Fermentas; NEB) and assembled by ligation or using NEBuilder HiFi Master Mix (NEB). All resulting plasmids were checked by sequencing. Cloning details are available upon request.

**TABLE 1:** Plasmids used in the study.

**Name of the vector**	**References**	**Parental vectors**	**Cloning sites/notes**
*CAG-bioSUMO3-T2A-BirA^*opt*^-T2A-GFPpuro*	Pirone et al., 2017	–	–
*CMV-CBX4-YFP*	This work	*pEYFP-N1*	*Eco*RI-*Sal*I (KAN); CBX4 generated by high-fidelity PCR
*CMV-SALL1-YFP*	Pirone et al., 2017	*pEYFP-N1*	*Eco*RI-*Sal*I (KAN); SALL1 generated by high-fidelity PCR
*CMV-SALL1ΔSUMO-YFP*	This work	*CMV-SALL1-YFP*	*Eco*RI-*Sal*I; mutants introduced by overlap extension PCR (KAN); K571R; K592R; K982R; K1086R
*CMV-SALL1ΔSIM-YFP*	This work	*CMV-SALL1-YFP*	*Eco*RI-*Sal*I; mutants introduced by overlap extension PCR (KAN); predicted SIMs mutated to AAAA; SIM71: VLIV; SIM195: VIIE; SIM254: ILLL; SIM1252: ISVI
*CMV-SALL1-2xHA*	This work	*CMV-SALL1-YFP*	EYFP exchanged for 2xHA using *Sal*I-*Not*I (KAN)
*CMV-SALL1^826^-2xHA*	This work	*CMV-SALL1(826)-YFP*	EYFP exchanged for 2xHA using *Sal*I-*Not*I (KAN)
CB6-HA-N	M. Way lab (CRUK, London)	*CB6*	CB6 has CMV promoter and confers neo selection; contains N-terminal HA epitope and MCS (AMP)
*CMV-EGFP-β-galactosidase*	This work	*pEGFP-N1*	LacZ subcloned from pIND/lacZ (Invitrogen)
*CB6-HA-SALL1*	This work	*CB6-HA-N*	*SALL1* from *CMV-SALL1-YFP*
*CB6-HA-SALL1ΔSUMO*	This work	*CB6-HA-N*	*SALL1* from *CMV-SALL1 ΔSUMO-YFP*
*CB6-HA-CBX4*	This work	*CB6-HA-N*	CBX4 from CMV-CBX4-YFP
CMV-SALL1-BirA*	This work	*CMV-SALL1-YFP*	Exchanged YFP for BirA*(BioID) by Sal1-Not1
CMV-Pc-BirA*	This work	CMV-SALL1-BirA*	*Drosophila Pc* (PCR amplified) exchanged for SALL1 using *Eco*R1-*Sal*1 (KAN); Pc source: Addgene #1927
CMV-CBX4-BirA*	This work	CMV-SALL1-BirA*	*CBX4* (PCR amplified) exchanged for *SALL1* using *Eco*R1-*Sal*1 (KAN)
CMV-BirAopt-2A-puro	Pirone et al., 2017	–	–
CMV-bioUB-2A-BirAopt-2A-puro	Pirone et al., 2017	–	–
LL-CMV-GFS-SALL1-IRES-puro	This work	LL-CMV-GFS-IRES-puro	SALL1 inserted into modified version of Lentilox3.7; expresses N-terminal GFP-FLAG-STREP tag
TripZ-SALL1-2xHA-puro	This work	*CMV-SALL1-2xHA; TRIPZ*	Inserted SALL1-2xHA amplicon into BshT1-Mlu1TRIPZ (Dharmacon)
*pcDNA3*	Invitrogen	–	–
Lenti-Cas9-blast vector	Addgene #52962	–	–
psPAX2	Addgene #12260	–	–
pMD2.G (VSV-G envelope)	Addgene #12259	–	–
*pEYFP-N1, pEYFP-C*	Clontech	–	–

*KAN or AMP indicate the antibiotic resistant cassette (kanamycin or ampicillin, respectively) in the vector for bacterial transformation.*

### Lentiviral Transduction

Lentiviral expression constructs were packaged using psPAX2 and pMD2.G in HEK 293FT cells, and cell culture supernatants were used to transduce HEK 293FT cells to generate stable cell populations expressing SALL1 (constitutive: LL-GFS-SALL1-IRES-puro; or inducible: TripZ-SALL1-2xHA-puro). Selection was performed using 1 μg/ml of puromycin.

### Bioinformatics Analyses

SUMOylation sites and SUMO-interacting motif (SIM) predictions were searched using SUMOplot,^1^ GPS-SUMO^2^ (Zhao et al., 2014), and JASSA programs (Beauclair et al., 2015). Sequence search and comparison was performed using BLAST.^3^ Alignments were performed using Clustal.^4^

### SUMOylation and Ubiquitination Assays in Cultured Cells

For the isolation of SUMOylated SALL1, one 10 cm dish of HEK 293FT cells was transfected with 7 μg of *CMV-SALL1-2xHA*, *CMV-SALL1ΔSUMO-2xHA*, and 3 μg of *CAG-bioSUMO3-T2A-BirA^*opt*^-T2A-GFPpuro* or *CAG-BirA^*opt*^-T2A-GFPpuro* as control. Isolation of SUMOylated protein was done according to previously reported methodology (Pirone et al., 2016, 2017).

For the ubiquitination assay of CBX4, one 10 cm dish was transfected with 5 μg of *CMV-SALL1-YFP*, *CMV-SALL1ΔSUMO-YFP*, *CMV-GFP-β-galactosidase*, *CMV-BirA-2A-puro*, *CMV-bioUB-2A-BirA-2A-puro*, or *CB6-HA-CBX4*. After transfection, medium was supplemented with biotin at 50 μM. Twenty-four hours after transfection, plates were treated with MG132 (10 μM, 12 h; Calbiochem). Transfected cells were collected after 48–72 h, washed three times with phosphate buffered saline (PBS) and resuspended in lysis buffer [0.5 ml/10 cm dish; 8 M urea, 1% SDS, 50 mM *N*-ethylmaleimide, 1× protease inhibitor cocktail (Roche) in 1× PBS]. Sonication was performed to reduce sample viscosity and samples were cleared by centrifugation at room temperature (RT). High-capacity NeutrAvidin-agarose beads (Thermo Scientific) were equilibrated and 30–60 μl suspension was used for incubation with extracts (12–18 h; RT; gentle agitation). Beads were subjected to stringent washes using the following washing buffers all prepared in 1× PBS (Franco et al., 2011): WB1 (8 M urea, 0.25% SDS); WB2 (6 M Guanidine-HCl); WB3 (6.4 M urea, 1 M NaCl, 0.2% SDS), WB4 (4 M urea, 1 M NaCl, 10% isopropanol, 10% ethanol, and 0.2% SDS); WB5 (8 M urea, 1% SDS); and WB6 (2% SDS). Samples were eluted in 50 μl of Elution Buffer (4× Laemmli sample buffer, 100 mM DTT) by two cycles of heating (5 min; 99°C), with vortexing in between. Beads were separated by centrifugation (18,000 × *g*, 5 min).

For the isolation of ubiquitinated endogenous CBX4 from cells lysates, 10 cm dishes were transfected with 5 μg of *CMV-SALL1-2xHA* plasmid or with pcDNA3 plasmid as control. After 48 h, cells were washed three times with 1× PBS and lysed in 500 μl of tandem ubiquitin binding entities (TUBEs) buffer [20 mM Phosphate buffer, pH 7.5 (Sigma), 2 mM EDTA (Sigma), 50 mM sodium fluoride (Sigma), 5 mM tetra-sodium pyro-phosphate (Sigma), and 10 mM β-glycerol 2-phosphate (Sigma)]. The buffer was filtered through a 0.22 μm membrane and stored at 4°C. Eighty microliters of the lysate were taken as input. Ubiquitinated material was isolated using TUBEs based on RAD23 Homolog A (RAD23A) ubiquitin binding domains fused to GST and expressed in bacteria (Hjerpe et al., 2009; Aillet et al., 2012). To eliminate proteins with binding affinity for the beads (Glutathione Sepharose 4B, GE Healthcare), lysates were incubated with 125 μg of GST bound to glutathione-agarose beads for 1 h at 4°C and centrifugated for 2 min at 1000 rpm. After washing GST-TUBES beads with cold 1× PBS twice, supernatants were added, incubated for 1 h at 4°C and centrifugated for 2 min at 1000 rpm. The supernatants were then removed and beads were washed three times with TUBEs buffer. The beads were washed three times with PBS-Tween 0.5% and twice with TUBEs buffer containing NaCl (0.5 M). Finally, the beads were resuspended in 50 μl of Boiling buffer (50 mM Tris–HCl pH 6.8, 10% glycerol, 2% SDS, Bromophenol Blue, 10% β-mercaptoethanol) warmed at 60°C before use.

### *In vitro* SUMOylation

Using PCR templates with incorporated 5′ T7 priming site +/− 3′ epitope-tags, SALL1-2xHA and CBX4 were transcribed/translated *in vitro* using the TNT^®^ Quick Coupled Transcription/Translation System (Promega) according to the manufacturer’s instruction and were then incubated in a buffer containing an ATP regenerating system [(50 mM Tris pH 7.5, 10 mM MgCl2, 2 mM ATP, 10 mM creatine phosphate (Sigma), 3.5 U/ml of creatine kinase (Sigma), and 0.6 U/ml of inorganic pyrophosphatase (Sigma)], 10 μg of SUMO1 or a combination of 5 μg of SUMO2 and SUMO3, 0.325 μg UBC9 and 0.8 μg of purified SAE1/2 (ENZO Life Sciences). SALL1 SUMOylation was checked adding 0.5–2 μl of *in vitro* transcribed/translated protein in the SUMOylation assay. Reactions were incubated at 30°C for 2 h and stopped by addition of SDS sample buffer.

### GFP-Trap Co-pulldown

HEK 293FT cells were plated at 25–30% confluence. Transient transfections were performed using calcium phosphate in a 10 cm dish with 5 μg of *CMV-CBX4-YFP*, *CMV-SALL1-YFP*, *CMV-SALL1ΔSUMO-YFP*, *CMV-SALL1ΔSIM-YFP*, *CMV-YFP*, *CMV-SALL1-2xHA*, *CMV-SALL1-826-2xHA*, *CB6-HA*, *CB6-HA-SALL1*, *CB6-HA-SALL1ΔSUMO* or *CB6-HA-CBX4* in complete medium. All steps after transfection were performed at 4°C. Two days after transfection, cells were washed three times with cold 1× PBS and detached from the dish with a scraper. Cells of 10 cm dishes were lysed by adding 1 ml of Lysis Buffer [25 mM Tris–HCl pH 7.5, 150 mM NaCl, 1 mM EDTA, 1% NP-40, 0.5% Triton X-100, 5% glycerol, protease inhibitors (Roche)] followed by incubation on a rotating wheel for 30 min at 4°C. Lysates were sonicated and spun down at 25,000 × *g* for 20 min. After saving 40 μl of supernatant (input), the rest of the lysate was incubated overnight with 30 μl of equilibrated GFP-Trap resin (Chromotek) in a rotating wheel. Beads were washed five times for 5 min each with washing buffer (25 mM Tris–HCl pH 7.5, 300 mM NaCl, 1 mM EDTA, 1% NP-40, 0.5% Triton X-100, 5% glycerol). Beads were centrifuged at 2000 × *g* for 2 min after each wash. For elution, samples were boiled for 5 min at 95°C in 2× Laemmli buffer.

### BioID Analysis of Interactions

Proximity interaction between CBX4 or Pc proteins to SALL1 was verified by the BioID method (Roux et al., 2013), consistent on fusing them to a promiscuous form of the enzyme BirA (BirA^∗^) and to isolate the biotinylated material by streptavidin−beads pulldowns. HEK 293FT cells were transfected with 5 μg of CMV-CBX4-BirA^∗^ or CMV-Pc-BirA^∗^ in combination with *CMV-SALL1-2xHA* or *CMV-SALL1826-2xHA*. After 24 h, the medium was supplemented with 50 mM of biotin. At 48 h, cells were washed three times in cold 1× PBS and collected in 1 ml of lysis buffer [8 M urea, 1% SDS, protease inhibitor cocktail (Roche) in 1× PBS]. Lysates were sonicated and cleared by centrifugation, incubated overnight with 40 μl of equilibrated NeutrAvidin-agarose beads (Thermo Scientific) and washed with WB1–6 as indicated in the ubiquitination protocol above. Elution was done as previously described using 50 μl of Elution Buffer (4× Laemmli sample buffer, 100 mM DTT) by two cycles of heating (5 min, 99°C), with vortexing in between. Beads were separated by centrifugation (18,000 × *g*, 5 min).

### Cycloheximide Assay

3 × 10^5^ HEK 293FT cells per well were plated in six-well plates. Four hours later, cells were transfected with 2 μg of *CMV-SALL1-YFP*, *CMV-SALL1ΔSUMO-YFP*, or *CMV-GFP-β-galactosidase* plasmid per well using the calcium phosphate method. Twenty-four hours after transfection, cells were treated with 50 μg of cycloheximide (CHX, 50 μg/ml) in combination or not with MG132 (10 μM) for different time points (0, 4, 8, or 16 h). Cells were lysed in RIPA buffer [150 mM NaCl, 1.0% NP-40, 0.5% sodium deoxycholate, 0.1% SDS, 50 mM Tris, pH 8.0, protease inhibitors (Roche)] and analyzed by Western blot.

### Western Blot

Samples were boiled at 95° for 5 min. Proteins were separated by SDS-PAGE (BioRad) and blotted using wet transfer to nitrocellulose membranes (0.45 μm pore; Cytiva). Membranes were blocked in 1× PBS with 0.1% Tween-20 (PBS-T) and 5% non-fat dry milk (blocking buffer) for 1 h and, for biotin detection, Casein Blocking Buffer 1× (Sigma #B6429). After that, membranes were incubated in blocking buffer for 1 h at RT or overnight at 4°C with the following primary antibodies: mouse monoclonal anti-HA (Sigma, 1:1000, #H3663), mouse monoclonal anti-β-Actin (Sigma, 1:1000, #A2228), mouse monoclonal anti-GFP (Roche, 1:1000, #11814460001), mouse monoclonal anti-SALL1 (R&D, 1:1000, #PP-K9814-00), rabbit polyclonal anti-CBX4 (Proteintech, 1:1000, #18544-1-AP), rabbit polyclonal anti-Avitag (GeneScript, 1:1000, #A00674), or rabbit monoclonal Vinculin (Cell Signaling, 1:1000, #13901S).

After three washes with PBS-T, the blots were incubated for 1 h with secondary antibodies: HRP-conjugated anti-mouse or anti-rabbit (1:5000, Jackson ImmunoResearch #115-035-062 or #111-035-045, respectively), HRP-conjugated anti-biotin (1:1000, Cell Signaling Technology #7075), HRP-conjugated anti-tubulin (1:5000, Proteintech #66031), or HRP-conjugated anti-GAPDH (1:5000, Proteintech #60004). Membranes were washed three times in PBS-T, developed using Clarity Western ECL substrate (Biorad) or Super Signal West Femto (Pierce), and chemiluminescent signals detected using a ChemiDoc camera system (Biorad). Quantification of bands was performed using Fiji software and normalized to Actin, GAPDH, or Vinculin levels, unless otherwise indicated. At least three independent blots were quantified per experiment.

### Immunostaining and Microscopy Analysis

For immunostaining and microscopy analysis, 50,000 cells per well were seeded in a 24 well-plate on 12 mm diameter round acid-washed sterile coverslips. U2OS cells were transfected with 2 μg of *CMV-SALL1-YFP*, *CMV-SALL1ΔSUMO-YFP*, or *pEYFP-C1*, 1.5 μg of *CMV-SALL1-YFP* or HEK 293FT_TripZ-SALL1-2xHA were used.

After 2 days cells were washed three times with cold 1× PBS, fixed in 4% paraformaldehyde (Santa Cruz) supplemented with 0.1% Triton X-100 in 1× PBS for 20 min at RT. Then, coverslips were washed three times with 1× PBS to remove the fixative. Blocking was performed in blocking buffer (1% BSA, 0.3% Triton X-100, 25 mM NaCl in 1× PBS) for 1 h at RT. Incubation with primary antibodies diluted in blocking solution was performed during 1 h at 37°C in a humidity chamber or overnight at 4°C. The following primary antibodies were used: rabbit polyclonal anti-SALL1 (1:200, Abcam #31905), mouse monoclonal anti-GFP (1:500, Roche #11814460001), mouse monoclonal anti-PML (Promyelocytic Leukemia Protein) (1:100, Santacruz #sc-966), mouse monoclonal anti-SC35 (Splicing Component, 35 KDa, also known as Serine and Arginine Rich Splicing Factor 2) (1:200, BD Pharmingen #556363), rabbit polyclonal anti-CBX4 (1:100, Proteintech #18544-1-AP), rabbit polyclonal anti-SUMO2/3 (1:100, Eurogentec #AV-SM23-0100), mouse monoclonal anti SUMO1 (1:100, Developmental Studies Hybridoma Bank, DSHB, #21C7), or mouse monoclonal anti-SUMO2 (1:100, DSHB #8A2). Endogenous SALL1 or SALL1-2xHA in HEK 293FT_TripZ-SALL1-2xHA cells were stained by a primary antibody against SALL1 (R&D, 1:100, #PP-K9814-00).

After incubation with the primary antibody, cells were gently washed three times with 1× PBS and then incubated with the secondary antibody in the dark for 1 h at RT. The secondary antibodies conjugated to fluorophores used were donkey anti-mouse or anti-rabbit Alexa Fluor 488, Alexa Fluor 568, or Alexa Fluor 647 (1:200, Molecular Probes). To visualize the nuclei, we incubated the cells with DAPI (1:15,000, Roche #10236276001) for 5 min at RT. Another three washes were performed to remove unbound secondary antibody. Finally, coverslips were mounted using Prolong Gold antifade reagent (Molecular Probes #P36930) and stored in the dark at 4°C.

Stained cells were visualized using an Upright Fluorescent Microscope Axioimager D1 or a Leica SP2 or SP8 confocal microscope with 63× objective. For the quantification of Pc bodies, Fiji software was used.

### Proximity Ligation Assays

U2OS cells were plated and transfected by PEI in six-well plates with 2 μg of *CMV-SALL1-2xHA* or *pcDNA3*. After 2 days, cells were transferred to an eight-well chamber slide (LabTek #177410) and allowed to attach for 12 h. Proximity ligation assay (PLA) was performed using the Duolink *In Situ* Red kit (Olink Bioscience; Gullberg et al., 2004; Söderberg et al., 2006) according to the manufacturer’s instructions. Primary antibodies used: mouse monoclonal anti-SALL1 (1:250, R&D Systems #PP-K9814-00); rabbit polyclonal anti CBX4 (1:100, Proteintech #18544-1-AP). Images were recorded on a Leica SP8 confocal microscope system using 488 and 561 nm wavelengths for excitation and a 63× lens for magnification, and were analyzed with the Leica confocal software, Adobe Photoshop, and ImageJ softwares.

### Reverse Transcription-Quantitative PCR Analysis

HEK 293FT cells transfected with 5 μg of *CMV-SALL1-YPF*, *CMV-SALL1ΔSUMO-YFP* or *CMV-GFP-β-galactosidase* plasmids, or HEK 293-TripZ-SALL1-2xHA_puro cells induced with different concentrations of doxycycline (dox), were used for reverse transcription-quantitative PCR (RT-qPCR) analysis. Forty-eight hours after transfection, or 72 h after induction, total RNA was obtained by using EZNA Total RNA Kit (Omega) and quantified using a NanoDrop spectrophotometry. cDNAs were prepared using the SuperScript III First-Strand Synthesis System (Invitrogen) using 1 mg of total RNA in 20 μl volume per reaction. qPCR was done using PerfeCTa SYBR Green SuperMix Low Rox (Quantabio). Reactions were performed in 20 μl, adding 5 μl of cDNA and 0.5 μl of each primer (10 μM), in a CFX96 thermocycler (BioRad) using the following protocol: 95°C for 5 min and 40 cycles of 95°C for 15 s, 56 or 62°C for 30 s and 72°C 20 s. Melting curve analysis was performed for each pair of primers between 65 and 95°C, with 0.5°C temperature increments every 5 s. Relative gene expression data were analyzed using the ΔΔCt method (Livak and Schmittgen, 2001). Reactions were carried out in duplicate and results were derived from at least three independent experiments, normalized to GAPDH and presented as relative expression levels. Primer sequences are listed in Table 2.

**TABLE 2:** Oligonucleotide sequences used for RT-qPCR.

**Name**	**Sequence**
hHoxa11_for	5′-AACGGGAGTTCTTCTTCAGCGTCT-3′
hHoxa11_rev	5′-ACTTGACGATCAGTGAGGTTGAGC-3′
hHoxb4_for	5′-AGGTCTTGGAGCTGGAGAAGGAAT-3′
hHoxb4_rev	5′-GGTGTTGGGCAACTTGTGGTCTTT-3′
hHoxb7_for	5′-AGACCCTGGAGCTGGAGAAAGAAT-3′
hHoxb7_rev	5′-ATGCGCCGGTTCTGAAACCAAATC-3′
hHoxb13_for	5′-TACGCTGATGCCTGCTGTCAACTA-3′
hHoxb13_rev	5′-AGTACCCGCCTCCAAAGTAACCAT-3′
hHoxc6_for	5′-AGGACCAGAAAGCCAGTATCCAGA-3′
hHoxc6_rev	5′-ATTCCTTCTCCAGTTCCAGGGTCT-3′
hHoxc10_for	5′-TGAAATCAAGACGGAGCAGAGCCT-3′
hHoxc10_rev	5′-TTGCTGTCAGCCAATTTCCTGTGG-3′
hHoxc12_for	5′-AGGGAACTCTCAGACCGCTTGAAT-3′
hHoxc12_rev	5′-AGAGCTTGCTCCCTCAACAGAAGT-3′
hHoxd13_for	5′-ATGTGGCTCTAAATCAGCCGGACA-3′
hHoxd13_rev	5′-AGATAGGTTCGTAGCAGCCGAGAT-3′
hGata4_for	5′-TCTCAGAAGGCAGAGAGTGTGTCA-3′
hGata4_rev	5′-GGTTGATGCCGTTCATCTTGTGGT-3′
hGAPDH_for	5′-CATGTTCGTCATGGGTGTGAACCA-3′
hGAPDH_rev	5′-AGTGATGGCATGGACTGTGGTCAT-3′
hSALL1_for	5′-GCTTGCACTATTTGTGGAAGAGC-3′
hSALL1_rev	5′-GAACTTGACGGGATTGCCTCCT-3′
hCBX4_for	5′-CATCGAGAAGAAGCGGATCCGCAAG-3′
hCBX4_rev	5′-CTGTTCTGGAAGGCGATCAGCAGCC-3′

### Statistical Analysis

Statistical analysis was performed using GraphPad 7.0 software. Data were analyzed by Shapiro-Wilk normality test and Levene’s test of variance. We used Mann–Whitney-*U* test or Unpaired *T*-test for comparing two groups and one-way ANOVA for more than two groups. *P*-values were represented by asterisks as follows: ^∗^*P*-value < 0.05; ^∗∗^*P*-value < 0.01; ^∗∗∗^*P*-value < 0.001; ^****^P-value < 0.0001. Differences were considered significant when *P* < 0.05.

## Results

### SALL1 Does Not Colocalize With CBX4 in Nuclear Bodies

In agreement with previous reports (Netzer et al., 2001; Kiefer et al., 2002; Sánchez et al., 2010; Abedin et al., 2011), we detected endogenous SALL1 in discrete domains in the nucleus of U2OS human osteosarcoma cells (Supplementary Figure 1A). Similar results were obtained in U2OS cells transfected with human SALL1-YFP (Supplementary Figures 1B,C). These SALL1 foci were reminiscent of Pc bodies, where PRC proteins, such as CBX4 accumulate. Thus, we hypothesized that SALL1 and CBX4 could colocalize in nuclear bodies.

**FIGURE 1:**
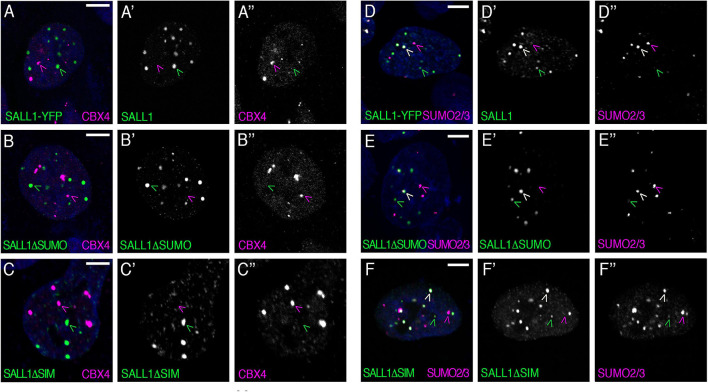
SALL1 and CBX4 do not colocalize in nuclear bodies. **(A–F)** Confocal images of U2OS cells showing expression of SALL1-YFP, SALL1ΔSUMO-YFP, or SALL1ΔSIM-YFP (green), and endogenous CBX4 (magenta in **A–C**) or endogenous SUMO2/3 (magenta in **D–F**). Nuclei were stained with DAPI. Black and white pictures show single green or magenta channels. Green arrowheads indicate SALL1 bodies, magenta arrowheads indicate Pc bodies **(A–C)**, or SUMO bodies **(D–F)** and white arrowheads indicate colocalization of SALL1 and SUMO2/3 **(D–F)**. Pictures were taken with a Leica DM IRE2 confocal microscope using a 63× objective. Scale bars indicate 5 μm.

To test this hypothesis, *SALL1-YFP* plasmid was transfected into U2OS cells, where endogenous CBX4 was visualized by immunofluorescence using anti-CBX4 specific antibodies. However, SALL1 and CBX4 were found to localize to different subsets of nuclear bodies (Figure 1A).

In order to further characterize the nature of CBX4 and SALL1 bodies, we explored their possible colocalization with SUMO. We transfected U2OS cells with SALL1-YFP and examined its localization, and that of endogenous CBX4, with SUMO using immunofluorescence. While CBX4 did not colocalize with SUMO1 or SUMO2/3 (Supplementary Figures 2A,B), a partial colocalization between SALL1 and SUMO proteins was observed: some of the SALL1 bodies clearly colocalized with SUMO1 and SUMO2/3, while other SALL1 bodies did not (Figure 1 and Supplementary Figure 2E). Conversely, some SUMO1 and SUMO2/3 bodies colocalized with SALL1, while others did not. These results fit with the well-known heterogenic nature of nuclear bodies (Zidovska, 2020). Neither CBX4, nor SALL1 colocalize with other nuclear factors, such as PML (Supplementary Figures 2C,H) or SC35 (Supplementary Figures 2D,I).

As shown previously, SALL1 undergoes SUMOylation in cells (Pirone et al., 2017), which might modulate its localization. To test this possibility, we generated a SALL1 SUMO mutant (SALL1ΔSUMO) by mutating four lysine residues (K571, K592, K982, and K1086) to arginine (Supplementary Figure 3A). These residues correspond to the four SUMOylation motifs conserved in vertebrates, predicted by SUMOplot and GPS-SUMO programs with highest scores (Supplementary Figures 3C,D) and the motif IKED (K982) being previously identified by proteomic analysis (Xiao et al., 2015; Hendriks and Vertegaal, 2016). As predicted, the SALL1ΔSUMO mutant lost the capacity to be SUMOylated in cells (Supplementary Figure 3B). Therefore, we considered SALL1ΔSUMO a SUMO-deficient mutant of SALL1. Interestingly, neither the lack of colocalization with CBX4, nor the partial colocalization with endogenous SUMO1 and SUMO2/3 were visibly altered when SALL1ΔSUMO-YFP was analyzed (Figures 1B,E and Supplementary Figure 2F). These results, indicating that the localization of SALL1 to a subset of SUMO bodies does not depend on its SUMOylation status, raised the possibility that SALL1 localization to these foci might be mediated by the presence of SIMs in this protein.

By analyzing the amino acid sequence of SALL1, we noted the presence of four high-scored SIMs (Supplementary Figures 3A,D). To investigate the role of these putative SIMs, we generated a SALL1ΔSIM version in which the four motifs were mutated to alanines. Remarkably, localization of SALL1 was unaffected by these mutations. Thus, SALL1ΔSIM-YFP readily localized to nuclear bodies, and partially colocalized with SUMO1 and SUMO2/3, but not with CBX4 (Figures 1C,F and Supplementary Figure 2G). The lack of colocalization between these two proteins in nuclear bodies prompted us to re-examine the interaction results obtained previously by mass spectrometry (MS).

### SALL1 Interacts With CBX4 in a SUMOylation-Independent Manner

**FIGURE 2:**
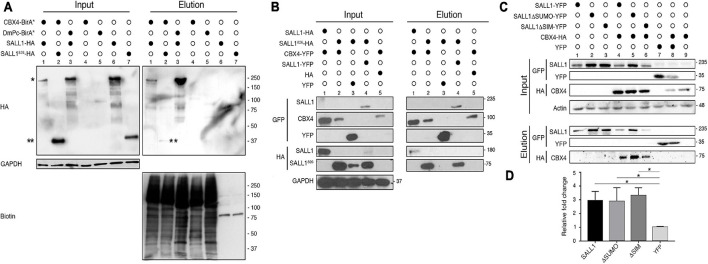
SALL1 interacts with CBX4 in a SUMOylation-independent manner. **(A)** Validation of the interaction between human SALL1 and human CBX4 or *Drosophila melanogaster* Pc proteins using BioID-based biotin pulldown in transfected HEK 293FT cells. In the Input panel, the relative expression of the HA-tagged SALL1 proteins (the full-length protein or a TBS-related truncation mutant) is shown. One asterisk indicates SALL1-HA, while two asterisks indicate SALL1^826^-HA. Negative controls (single expression of each individual protein) are shown in lanes 4–7. Anti-GAPDH was used as loading control. As shown in the Elution panel, CBX4-BirA* interact preferentially with full-length SALL1-HA (lane 1). Anti-biotin blot shows the efficiency of the different pulldowns. **(B)** Validation of the interaction between SALL1 and CBX4 using GFP-Trap. The Input panel shows the expression of epitope-tagged SALL1 and CBX4 proteins in transfected HEK 293FT cells. YFP alone and HA empty vector were used as controls. Lanes 1 and 2 of the Elution panel, show that CBX4 interacts with SALL1 full length and the truncated form. **(C)** SUMO-related SALL1 mutants interact with CBX4. Western blot analysis of proteins extracted from HEK 293FT cells transfected with the indicated plasmids. Pulldowns were performed using GFP-Trap. As shown in the Elution panel (lanes 4, 5, and 6), interaction between CBX4 and WT SALL1 or SALL1 mutants was readily detected in all blot images. **(D)** Graph showing that CBX4 levels increase when co-expressed with WT SALL1-YFP, SALL1ΔSUMO-YFP, or SALL1ΔSIM-YFP. The intensity of CBX4 bands in blots was quantified using ImageJ, normalized to b-Actin and reported as fold change relative to the YFP alone control. The mean plus SEM of three independent experiments is plotted. *P*-values were calculated using Mann–Whitney test. *P-value < 0.05. **(A–C)** Antibodies used are indicated to the left. Molecular weight markers are indicated to the right in KDa.

Previous MS results suggested that CBX4 could interact with full length SALL1 (Bozal-Basterra et al., 2018). We checked whether we could detect the CBX4-SALL1 interaction using CBX4-BioID. HEK 293FT cells were transfected with CBX4 fused to a promiscuous variant of the BirA biotin ligase (CBX4-BirA^∗^) together with either full length SALL1-2xHA or the truncated form of SALL1^826^-2xHA, causative of TBS. After pulldown using NeutrAvidin beads, the eluates were analyzed by Western blot. As shown in Figure 2A, CBX4 was in close proximity to both the full length and the truncated SALL1 forms (elution panel, lanes 1 and 2). *Drosophila* Pc (DmPc-BirA, the fly CBX4 homolog; lane 3) is also able to interact with full-length SALL1-HA.

We further confirmed the interaction between SALL1 and CBX4 by using pulldown experiments. CBX4-YFP was transiently overexpressed in HEK 293FT together with SALL1-2xHA or SALL1^826^-2xHA, and GFP-Trap-based pulldown assays were carried out. SALL1-YFP was used as a positive control, since it is known to bind to the truncated mutant. As shown in Figure 2B, CBX4-YFP interacted both with full length and truncated SALL1 (elution panel, lanes 1 and 2).

SALL1 post-translational modifications could affect its interaction with other proteins. In this regard, SALL1 SUMOylation might be particularly relevant for its interaction with CBX4, which contain SIM domains (Merrill et al., 2010). In order to test whether SUMOylation could have a role in SALL1 binding to CBX4, we analyzed the SALL1ΔSUMO capability to interact with CBX4 (Figure 2C). WT SALL1-YFP and SALL1ΔSUMO-YFP were transiently transfected in HEK 293FT cells together with CBX4-HA (lanes 4 and 5, respectively). A GFP-Trap pulldown was performed and analyzed by Western blot. Our results show that the SUMOylation-deficient SALL1 mutant was still able to interact with CBX4 (elution panel, compare lanes 4 and 5). No appreciable differences were noted between WT SALL1 and SALL1ΔSUMO in their ability to interact with CBX4.

On the other hand, since CBX4 is known to be SUMOylated *in vitro* (Kagey et al., 2003; Merrill et al., 2010), we tested whether the predicted SIMs in SALL1 could have a role in its interaction with CBX4. As shown in Figure 2C (elution panel, compare lanes 4 and 6) SALL1 WT and SALL1ΔSIM showed similar capacity to bind CBX4. While differences in the intensity of CBX4 signals between SALL1 WT, SALL1ΔSUMO and SALL1ΔSIM can be observed, these differences were mostly due to the expression levels of the YFP-tagged SALL1 proteins. For example, the higher expression levels of SALL1ΔSUMO compared to SALL1 WT are most likely directly related to the higher levels of CBX4-HA detected in the pulldown.

In summary, these results confirm SALL1/CBX4 interaction, and show that neither SALL1 SUMOylation, nor its predicted SIM motifs are necessary for binding to CBX4 in our experimental setting.

### SALL1 and CBX4 Interact in the Nucleoplasm

Both proteins localize to the nucleus, with non-overlapping enrichment in nuclear bodies, so we thought that the SALL1-CBX4 interaction might occur in the nucleoplasm where weaker immunofluorescence signals can be observed (Supplementary Figure 4). In order to explore this possibility, we decided to apply the PLA, a technique that allows the detection of protein–protein interactions *in situ*.

**FIGURE 3:**
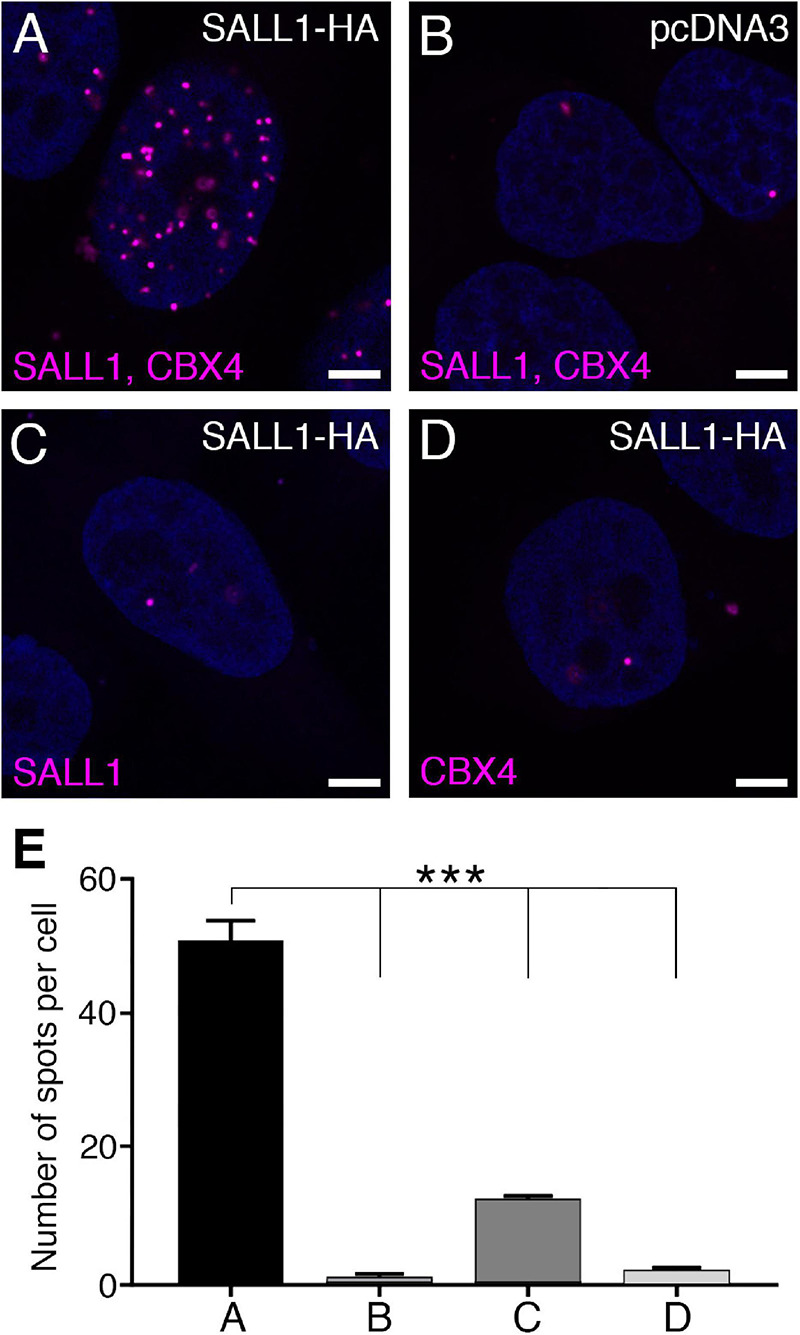
SALL1 and CBX4 interact in the nucleoplasm. **(A–D)** Confocal pictures of a proximity ligation assay (PLA) showing *in situ* interaction of SALL1 and CBX4 in the nucleus of U2OS cells, visualized as magenta spots. Cells were transfected with SALL1-HA or with the empty pcDNA3 vector as negative control. Antibodies used in the assay are indicated in magenta. Panel **A** shows SALL1 and CBX4 interaction, while panels **B–D** are negative controls. **(E)** Quantification of PLA signals per cell as in **A–D**. Bars represent mean plus SEM of three independent experiments. *P*-values were calculated using one-way ANOVA test. ***P-value < 0.001.

U2OS cells were transfected with *CMV-SALL1-2xHA* or with an empty *pcDNA3* vector as negative control, and anti-SALL1 and anti-CBX4 antibodies were used to perform PLA (Figures 3A–E; Söderberg et al., 2006; Matic et al., 2010). The signal from each detected pair of PLA probes is visualized as a fluorescent spot. Our analysis of the number of spots revealed an interaction between SALL1 and CBX4 in the nucleus (Figures 3A,E). Combined with the SALL1/CBX4 localization analyses described above, these results suggest that the interaction between SALL1 and CBX4 takes place most probably in the nucleoplasm instead of in nuclear bodies.

### SALL1 Post-transcriptionally Increases the Levels of CBX4

Considering previous evidence that SALL1 can be SUMOylated and that CBX4 can act as an E3 ligase to increase SUMOylation of several substrates (Kagey et al., 2003; Li et al., 2007; MacPherson et al., 2009; Ismail et al., 2012; Pelisch et al., 2012; Chen et al., 2018), we hypothesized that the SALL1/CBX4 interaction could drive SALL1 SUMOylation. However, our *in vitro* SUMOylation assays in the presence of SUMO1 or SUMO2/3 showed that the SUMOylated form of SALL1 did not vary in a statistically significant manner when different amounts of CBX4 were added to the reaction (Supplementary Figure 5).

**FIGURE 4:**
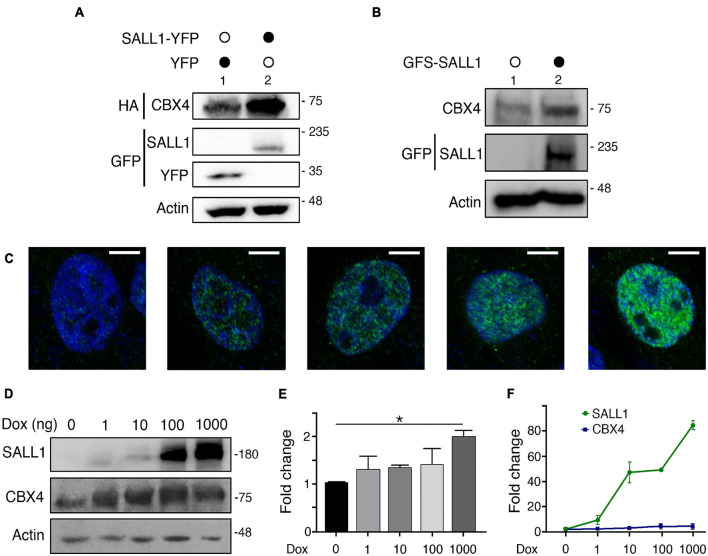
SALL1 influences the levels of CBX4. **(A)** Western blot showing protein levels of CBX4-HA when co-expressed with SALL1-YFP or YFP alone in HEK 293FT cells. Actin expression was used as loading control. **(B)** Western blot showing expression levels of endogenous CBX4 protein in parental HEK 293FT cells (lane 1) or in HEK 293FT cells stably expressing GFS-SALL1 (lane 2). **(C)** Confocal microscopy images showing inducible expression SALL1-2xHA in HEK 293FT_TripZ-SALL1-2xHA cells. Cells were treated with different concentrations of doxycycline (Dox) to induce SALL1 expression as indicated. SALL1-2xHA was detected using anti-SALL1 primary antibody (green). Cell nuclei were stained with DAPI (blue). **(D)** Western blot analysis showing expression levels of endogenous CBX4 in HEK 293FT_TripZ-SALL1-2xHA cells treated with increasing concentrations of Dox. **(E)** Quantification of the expression levels of endogenous CBX4 in HEK 293FT_TripZ-SALL1-2xHA cells treated with increasing concentrations of Dox. Three independent experiments as the one shown in **D** were performed. The intensity of CBX4 bands was quantified using ImageJ, and the values were normalized to the levels of Actin. *P*-value was calculated using one-way ANOVA test. *P-value < 0.05. **(F)** RT-qPCR analysis of *SALL1* and *CBX4* mRNA expression in HEK 293FT_TripZ-SALL1-2xHA cells treated with increasing concentrations of Dox. SALL1 and CBX4 expression were normalized using *GAPDH* expression and shown as fold change relative to untreated control. **(A,B,D)** Molecular weight markers are shown to the right in KDa. Antibodies were used as indicated to the left. **(E,F)** The mean plus SEM of at least three independent experiments is shown.

These results suggested that CBX4 does not function as a SUMO E3 ligase for SALL1 in this experimental settings, leaving the question of what could be the biological outcome of the interaction between these proteins unanswered. Intriguingly, while performing the experiments to validate the SALL1-CBX4 interaction, we had noticed that the levels of CBX4 were higher in cells co-transfected with SALL1 proteins (SALL1 WT, SALL1ΔSUMO, or SALL1ΔSIM) than in control cells co-expressing YFP (Figure 2B, lanes 1 vs. 5; Figure 2C, lanes 4, 5, and 6 vs. 8 and 9). This observation was supported by a quantitative analysis of the immunoblot results (Figure 2D), and was further confirmed using a transient co-expression experiment in HEK 293FT cells. In this experiment, Western blot analysis revealed higher levels of CBX4-HA in cells co-expressing SALL1-YFP than in cells co-expressing YFP alone (Figure 4A).

In order to discard any potential artifact due to the transient overexpression conditions, we generated two HEK 293FT-derived cell lines stably expressing SALL1. On one hand, we generated a HEK 293FT cell line constitutively expressing a GFS (GFP-Flag-Strep)-tagged version of SALL1 at levels moderately increased over the endogenous SALL1. Western blot analysis showed increased levels of endogenous CBX4 in HEK 293FT_GFS-SALL1 cells compared with parental HEK 293FT cells (Figure 4B). On the other hand, we used the inducible lentiviral vector TripZ to generate the HEK 293FT_TripZ-SALL1-2xHA cell line (see section “Materials and Methods”). This vector, based on the Tet-On system, allowed us to induce the expression of SALL1-2xHA in a doxycycline dependent manner, while preserving the expression of endogenous SALL1. As verified by immunofluorescence analysis (Figure 4C), increasing concentrations of doxycycline (1 ng/ml, 10 ng/ml, 0.1 μg/ml, or 1 μg/ml), lead to a progressive increment of the SALL1 expression in HEK 293FT_TripZ-SALL1-2xHA cells. The levels of endogenous CBX4 protein were analyzed in these cells using Western blot (Figures 4D,E). Quantification of three independent experiments showed that CBX4 levels were significantly increased when the cells were treated with 1 μg/ml of doxycycline compared to untreated cells (Figure 4E).

Since SALL1 is a transcription factor, we wondered whether the increased CBX4 levels described above could be due to SALL1-mediated transcriptional activation of CBX4 expression, potentially in an indirect way, as SALL1 is mostly described as a transcriptional repressor. We tested this possibility using the inducible HEK 293FT_TripZ-SALL1-2xHA cell model. SALL1 and CBX4 mRNA expression was analyzed by RT-qPCR) in control or doxycycline-treated cells. As expected, SALL1 mRNA expression increased in a doxycycline-dependent manner (Figure 4F). However, CBX4 mRNA expression levels did not vary significantly.

Altogether these results demonstrate that increasing levels of SALL1 are correlated with increasing CBX4 protein levels and, importantly, that this effect occurs at a post-transcriptional level.

### SALL1 Stabilizes CBX4 Avoiding Its Degradation *via* the Proteasome

Different mechanisms may contribute to increase the levels of a given protein, including changes in subcellular localization, solubility, or alteration in protein stability due to reduced degradation. The results described above led us to test the hypothesis that SALL1 could stabilize CBX4.

To this end, we analyzed the half-life of CBX4 by using a time-course experiment with CHX. HEK 293FT cells were transfected with WT SALL1-YFP, SALL1ΔSUMO-YFP, or GFP-β-gal and treated with 50 μg/ml of CHX in presence or absence of 10 μM of the proteasome inhibitor MG132. Cells were collected at different time points (0, 4, 8, and 16 h after initiation of treatment) and the levels of endogenous CBX4 were analyzed by Western blot.

**FIGURE 5:**
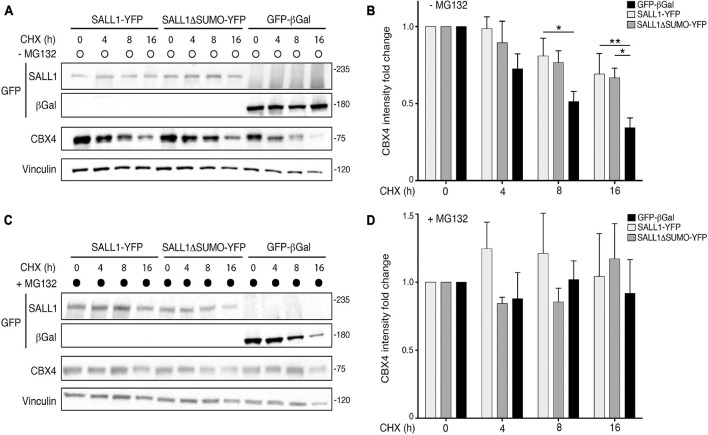
SALL1 stabilizes CBX4 protein. **(A,C)** Western blot analysis of cycloheximide (CHX) chase experiments performed in HEK 293FT cells transfected with *SALL1-YFP*, *SALL1ΔSUMO-YFP*, or *GFP-β-gal*. Cells were treated with 50 μg/ml of CHX in the absence **(A)** or presence **(C)** of 10 μM of the proteasome inhibitor MG132. Cells were collected at different time points (0, 4, 8, and 16 h after initiation of treatment) and endogenous CBX4 levels were analyzed by Western blot. Vinculin was used as loading control. Molecular weight markers are shown to the right in KDa. Antibodies were used as indicated to the left. **(B,D)** CBX4 levels were quantified after CHX treatment alone **(B)** or in combination with MG132 **(D)**, normalized to Vinculin, and data from six different independent experiments were pooled together. Graphs show mean plus SEM. *P*-values were calculated using one-way ANOVA test. *P-value < 0.05; **P-value < 0.01.

As shown Figure 5A, the levels of CBX4 began to decrease after 4 h of CHX treatment in cells expressing GFP-β-gal. However, in SALL1 WT or SALL1ΔSUMO-transfected cells the reduction in CBX4 levels was slower than in control cells. Quantification of six independent experiments is shown in Figure 5B. When cells were co-treated with CHX and MG132 (Figure 5C), proteasome degradation was inhibited and CBX4 levels did not decline at 4 h. Consequently, as shown in the Western blot quantification, no significant differences in the CBX4 levels were observed between cells transfected with SALL1, SALL1ΔSUMO, or control (Figure 5D). Overall, these results show that CBX4 protein is more stable in presence of SALL1 or SALL1ΔSUMO, and that degradation of CBX4 occurs through the ubiquitin proteasome system (UPS). Therefore, we concluded that SALL1 stabilizes CBX4 protein slowing down its degradation *via* the proteasome, and that SUMOylation of SALL1 seems not to be essential for CBX4 stabilization.

### SALL1 Influences CBX4 Ubiquitination

**FIGURE 6:**
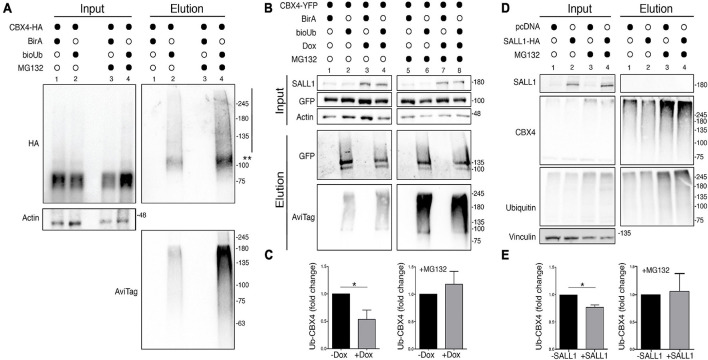
CBX4 ubiquitination is reduced in presence of SALL1. **(A)** Western blot analysis of HEK 293FT cells transfected with *CBX4-HA* together with *CMV-BirA-2A-bioUb* or *BirA* as a negative control. Cells were treated with 50 μM of biotin in the presence or absence of 10 μM MG132. Protein lysates were subjected to pulldown with streptavidin beads and the results were analyzed by Western blot. Two asterisks indicate monoubiquitinated CBX4-HA protein and the vertical line indicates the polyubiquitination smear. **(B)** Western blot analysis of HEK 293FT_TripZ-SALL1-2xHA cells transiently transfected with *CBX4-YFP* together with *BirA-2A-bioUb* or *BirA* as control. The cells were treated or not with 1 μg/ml of doxycycline (Dox), in presence or absence of 10 μM of MG132. Protein lysates were incubated with streptavidin beads to isolate bioUb conjugated proteins and results were analyzed by Western blot. β-Actin was used as loading control. **(C)** The levels of ubiquitinated CBX4-YFP in Dox induced and not induced cells, in presence (right panel) or absence (left panel) of MG132, were quantified and normalized to the CBX4 levels in the input. **(D)** Western blot analysis of endogenous CBX4 in HEK 293FT cells transfected with *CMV-SALL1-2xHA* (lanes 2 and 4) or with pcDNA3 control plasmid (lanes 1 and 3), in presence (lanes 3 and 4) or absence (lanes 1 and 2) of 10 μM MG132. **(E)** Quantification of ubiquitinated CBX4 in the elution panel normalized to the CBX4 levels in the input, in cells expressing or not SALL1-HA, in presence (right panel) or absence (left panel) of MG132. **(A,B,D)** Molecular weight markers are shown to the right in KDa. Antibodies were used as indicated to the left. **(C,E)** Graphs represent mean plus SEM. *P*-values were calculated on *n* = 4 using Mann–Whitney test. *P-value < 0.05.

Previous reports have shown that CBX4 is ubiquitinated to mediate its degradation through the proteasome (Ning et al., 2017). To investigate a potential relationship between SALL1 expression and CBX4 ubiquitination, we used the bioUb system (Pirone et al., 2017). First, we tested the efficiency of this system to detect the ubiquitinated fraction of CBX4. We transiently transfected HEK 293FT cells with CBX4-HA together with BirA-2A-bioUb or BirA as control. Cells were treated with biotin in presence or absence of the proteasome inhibitor MG132. Protein lysates were processed for bioUb assay (see section “Materials and Methods”) and results were analyzed by Western blot (Figure 6A). Ubiquitinated CBX4 is shown in the elution panel. A band above 100 KDa and a high molecular weight smear, both consistent with ubiquitinated forms of CBX4, are visible. As expected, the levels of ubiquitinated CBX4 increased in presence of the proteasome inhibitor MG132. Anti-Avitag antibodies detecting bioUb also showed an increase in the general ubiquitination levels in presence of MG132, as shown in the elution panel. These results confirmed the modification of CBX4 by ubiquitination and its degradation *via* UPS.

Next, to test whether SALL1 could increase CBX4 stability by impairing its ubiquitination and subsequent proteasomal degradation, we studied CBX4 ubiquitination in the inducible HEK 293FT_TripZ-SALL1-2xHA cells. These cells were transiently transfected with *CBX4-YFP* together with *BirA-2A-bioUb* or *BirA* as control. The cells were treated or not with 1 μg/ml of doxycycline to induce SALL1 expression, in the presence or absence of 10 μM MG132. Protein lysates were processed for bioUb assay, and the results were analyzed by Western blot (Figure 6B). A statistically significant reduction of CBX4 ubiquitination was observed in presence of high levels of SALL1 (Figures 6B,C, in the elution panel compare lane 4 with lane 2). However, in the presence of MG132, no significant differences were appreciated between induced and not induced cells (Figure 6C, in the elution panel compare lanes 6 and 8).

To further analyze the ubiquitination of endogenous CBX4, we transiently expressed SALL1-2xHA or pcDNA3 as a control in HEK 293FT cells. After lysis, total ubiquitinated material was isolated from the cells by pulldown using TUBES (see section “Materials and Methods”), and analyzed by Western blot (Figures 6D,E). In presence of SALL1, the levels of ubiquitinated CBX4 were reduced when compared with cells transfected with the control plasmid (elution panel, compare lanes 1 and 2). No significant differences were appreciated when cells were treated with MG132 (elution panel, lane 4 vs. lane 3). Quantification of ubiquitinated CBX4 in relation to the CBX4 input in shown in Figure 6E. Taken together, these results indicated that SALL1 is able to stabilize CBX4 protein by reducing its ubiquitination and subsequent degradation *via* the UPS.

### SALL1 Modulates the Number and Size of CBX4-Containing Pc Bodies, as Well as the Expression of CBX4 Target Genes

**FIGURE 7:**
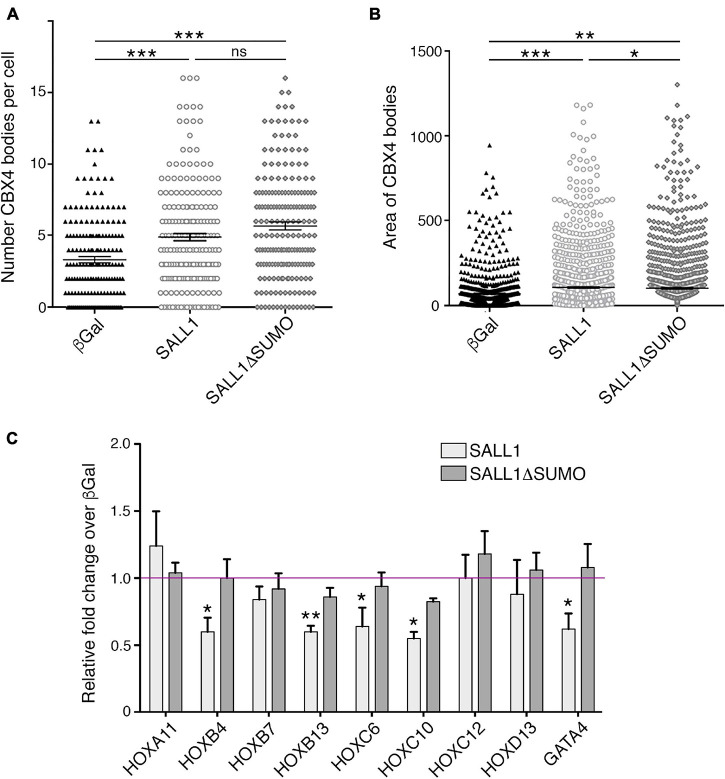
SALL1 expression increases the number and size of CBX4-containing Pc bodies and enhances downregulation of CBX4 targets. **(A,B)** Graphs represent the number of CBX4-containing Pc bodies **(A)** and their mean area in pixels quantified using Fiji software **(B)** in U2OS cells expressing SALL1-YFP, SALL1ΔSUMO-YFP, or GFP-β-gal as a negative control. **(C)** Graph showing the mRNA expression levels of several CBX4 target genes in HEK 293FT cells expressing SALL1-YFP, SALL1ΔSUMO-YFP, or GFP-β-gal as control. Data shown correspond to the mean plus SEM of at least five independent RT-qPCR experiments. Gene expression data were normalized to *GAPDH* and are shown as relative fold change over β-Gal expressing cells (magenta line). *P*-values were calculated using one-way ANOVA test. *P-value < 0.05; **P-value < 0.01; ***P-value < 0.001.

Although SALL1 does not colocalize with CBX4 in Pc bodies, the finding that SALL1 modulates CBX4 protein levels prompted us to investigate a potential effect of SALL1 expression on CBX4-containing Pc bodies. We transiently transfected SALL1-YFP or its mutant SALL1ΔSUMO-YFP in U2OS cells. GFP-β-gal was transfected as control. Transfected cells were stained with a specific CBX4 primary antibody and the number and area of CBX4-containing Pc bodies were examined in more than 100 cells per condition (Supplementary Figure 6). Using confocal microscopy and image analysis with Fiji software (Figures 7A,B), we observed that Pc bodies were significantly larger and more abundant in cells expressing SALL1 or SALL1ΔSUMO than in cells expressing β-Gal. No significant differences in the number of bodies were observed between cells expressing SALL1 and SALL1ΔSUMO. However, the area of the Pc bodies was significantly smaller in SALL1ΔSUMO compared to SALL1 transfected cells. These results revealed that SALL1 SUMOylation status does not influence the increase in the number of Pc bodies, but it may influence their size.

Finally, since SALL1 increases CBX4 protein levels, as well as the size and number of Pc bodies, and increased formation of Pc bodies may lead to stronger transcriptional repression of several PRC1 target genes (Gonzalez et al., 2014; Soshnikova, 2014; Cheutin and Cavalli, 2018), we hypothesized that SALL1 overexpression could lead to a stronger transcriptional repression of CBX4 targets, including HOX genes.

To test this possibility, HEK 293FT cells were transiently transfected with SALL1-YFP, SALL1ΔSUMO-YFP, or GFP-β-gal as control, and the expression levels of several direct CBX4 target genes (*HOXA11*, *HOXB4*, *HOXB7*, *HOXB13*, *HOXC6*, *HOXC10*, *HOXC12*, *HOXD13*, and *GATA4*) were analyzed by RT-qPCR. Significant differences in the expression of *HOXB4*, *HOXB13*, *HOXC6*, *HOXC10*, and *GATA4* were observed between wild-type SALL1 and β-Gal expressing control cells (Figure 7C). However, no significant differences were observed between SALL1ΔSUMO-transfected cells and control cells.

Taken together, these results indicate that high SALL1 levels modulate the transcriptional repression capacity of CBX4 on some of its target genes. Interestingly, SUMOylation of SALL1 seemed to be necessary for this transcriptional effect.

## Discussion

In this work, we have confirmed that SALL1 and CBX4 proteins interact with each other. Although both proteins can be SUMOylated and contain validated [CBX4 (Merrill et al., 2010)] or predicted (SALL1) SIM motifs, our results suggest that the SALL1/CBX4 interaction does not depend on the SUMOylation status of SALL1, nor the mutation of its putative SIMs. We note the possible contribution of the endogenous SALL1 to the interaction, as dimers with the endogenous WT SALL1 and exogenous mutants could be formed, bridging the interaction of mutant SALL1 with CBX4.

Neither SALL1 WT nor the SALL1ΔSUMO or SALL1ΔSIM mutant forms showed colocalization with CBX4 in Pc bodies, a subset of nuclear bodies that have been defined as centers of chromatin regulation for transcriptional repression of target genes (Entrevan et al., 2016). This observation indicates that the SALL1-CBX4 interaction does not occur in this specific cellular compartment. Despite this, we demonstrate that SALL1, as well as its SUMOylation-deficient mutant form, increase the number and size of CBX4-containing Pc bodies. We speculate that a dynamic and transitory interaction with SALL1 in the nucleoplasm may indirectly influence Pc body formation by altering CBX4 levels. In fact, we demonstrated that SALL1 stabilizes and increases CBX4 protein levels in a post-translational manner, reducing its ubiquitination with subsequent reduction of its degradation *via* the proteasome.

Different hypothetical scenarios could explain the SALL1-mediated stabilization of CBX4. As a transcriptional repressor, SALL1 could inhibit the transcription of ubiquitin E3 ligase(s) involved in CBX4 modification or could facilitate the binding and/or the recognition of CBX4 by DUBs (Ning et al., 2017). Interestingly, SALL1 was found to interact with members of the UPS, which might disrupt CBX4 homeostasis (Bozal-Basterra et al., 2018). Importantly, we show that high SALL1 levels increase CBX4-mediated transcriptional repression of some of its target genes. Although SUMOylation of SALL1 does not seem to affect its ability to regulate CBX4 protein levels, it seems to be important for SALL1 to modulate CBX4 transcriptional repression activity: only when SALL1 is SUMOylated, the recruitment of CBX4 on the chromatin results in a functional effect. In a speculative scenario, one possible explanation of these results could be the involvement of a third component. For instance, SUMOylation of SALL1 could facilitate the simultaneous interaction with other members of the PRC1, such as RING1 or PHC1. Interestingly, those factors were also found as possible SALL1 interactors in the proximity proteomics analysis that hinted initially to a possible SALL1/CBX4 interaction (Bozal-Basterra et al., 2018). Otherwise, SUMOylation of SALL1 could facilitate the interaction of CBX4 with co-factors required for gene repression (Cheng et al., 2014).

**FIGURE 8:**
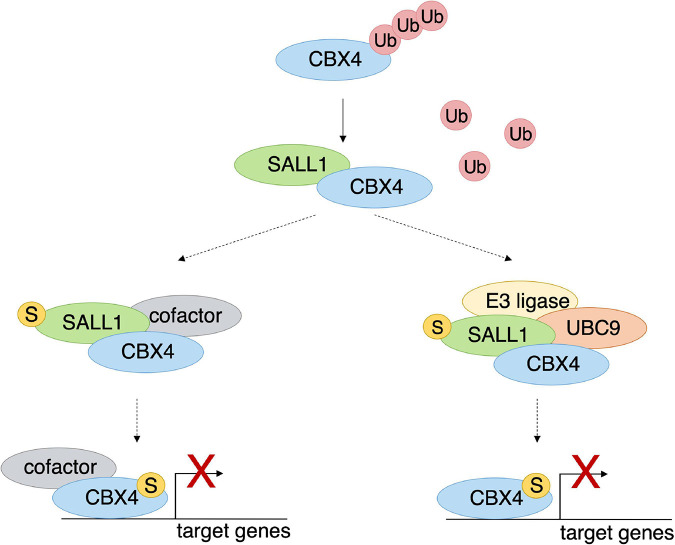
SALL1 influences regulation of CBX4 target genes. Hypothetical model showing speculative scenarios whereby SALL1 could influence CBX4-mediated regulation of target genes. Binding to SALL1 (SUMOylated or non-SUMOylated) could stabilize CBX4 by interfering with its ubiquitination and its consequent degradation by the proteasome. CBX4 stabilization entails an increment of its protein levels and its accumulation in Pc bodies. Binding to SUMOylated SALL1 increases CBX4-mediated transcriptional repression of its target genes. At least two non-exclusive hypothetical mechanisms might underlie this effect. Under one hypothetical scenario (left side), it could be due to the concurrent recruitment of other essential cofactors. In another hypothetical scenario (right side), SUMOylated SALL1 could increase CBX4 transcriptional repression by facilitating its SUMOylation through recruitment of SUMOylation machinery components. Discontinuous arrows indicate speculative events that have not been proven experimentally.

These highly speculative hypotheses can be summarized into the model shown in Figure 8. SALL1 (in its unmodified or SUMOylated form) would interact with CBX4. This interaction would result in less ubiquitination of CBX4 with its consequent stabilization (Figure 8). Thus, CBX4 would be recruited on chromatin, where it would act as a transcriptional repressor of its target genes. In its SUMOylated form, SALL1 could interact, not only with CBX4, but also with repression cofactors or other components of PRC1, which could be recruited on chromatin along with CBX4 (Figure 8, left). The recruitment of transcriptional cofactor(s), or various components of PRC1, would result in the activation of the multiprotein complex with consequent repression of the target genes.

In an alternative hypothesis, SUMOylated SALL1 could enhance CBX4 repression capacity by facilitating its SUMOylation. The SUMOylation of CBX4 is known to be necessary for its repression activity on the chromatin (Kang et al., 2010). We observed that, in the presence of high levels of SALL1, the SUMOylation of CBX4 increased (data not shown). However, this was probably the result of increasing the total levels of the protein. In addition, SALL1 was demonstrated to interact with UBC9 and SUMO1 in a yeast two-hybrid system (Netzer et al., 2002). Interestingly, some members of the SUMOylation pathway were also found in the proximity proteomics analysis of SALL1 (Bozal-Basterra et al., 2018). In this alternative hypothetical scenario, once SUMOylated SALL1 promotes CBX4 stabilization impairing its ubiquitination, it would be able also to promote CBX4 SUMOylation by recruiting an E3 SUMO ligase or other components of the SUMOylation machinery (Figure 8, right). In this regard, the K224 residue involved in CBX4 SUMOylation, and the adjacent K209 and K247 residues were predicted as putative ubiquitination sites by UbPred.^5^ This raises the interesting possibility that modification of CBX4 by ubiquitin and SUMO would be mutually exclusive events. Whether this is the case, and whether SALL1 is involved in this regulation, would require further investigation.

Additional experiments are necessary to further test the non-mutually exclusive hypotheses for SALL1-mediated regulation of CBX4. Our results suggest that SALL1 plays an important role in the control of the expression of key developmental genes through the post-transcriptional regulation of CBX4. Where and when this regulation takes place *in vivo* during development deserves further investigation.

## Data Availability Statement

The original contributions presented in the study are included in the article/Supplementary Material, further inquiries can be directed to the corresponding author/s.

## Author Contributions

IG, LP, JS, and RB designed the experiments and analyzed the data. IG, LP, JR, JS, and RB wrote the manuscript. IG, LP, VM, EL, CP, VL, EG-L, MG-L, and OB-G developed the experimental protocols, performed the experiments, and analyzed the data. ACOV, AA, JR, and MSR provided the scientific resources. All authors contributed to the article and approved the submitted version.

## Conflict of Interest

VL is employed by the company Viralgen Vector Core. The remaining authors declare that the research was conducted in the absence of any commercial or financial relationships that could be construed as a potential conflict of interest.

## Publisher’s Note

All claims expressed in this article are solely those of the authors and do not necessarily represent those of their affiliated organizations, or those of the publisher, the editors and the reviewers. Any product that may be evaluated in this article, or claim that may be made by its manufacturer, is not guaranteed or endorsed by the publisher.

## Footnotes

1
http://www.abgent.com/sumoplot

2
http://sumosp.biocuckoo.org

3
http://blast.ncbi.nlm.nih.gov/Blast.cgi/

4
https://www.ebi.ac.uk/Tools/msa/clustalo/

5
http://www.ubpred.org/

## Supplementary Material

Supplementary Figure 1 SALL1 localizes to nuclear bodies. Endogenous SALL1 **(A)** and transiently expressed SALL1-YFP **(B)** localize to nuclear bodies in U2OS cells. In contrast, YFP alone, used as a control, shows a homogenous distribution in the nucleus and cytoplasm **(C)**. Pictures were taken with an AxioD Fluorescent microscope using 100× objective. Scale bars indicate 5 μm.

Supplementary Figure 2 Characterization of CBX4 and SALL1 nuclear bodies. **(A–D)** Endogenous CBX4 (green) does not colocalize with SUM2/3, SUMO1, nor PML bodies or with SC35 (magenta) in U2OS cells. **(E–I)** SALL1-YFP (green) partially colocalizes with endogenous SUMO1 (magenta) in U2OS cells **(E)**. Similar results were obtained for the SALL1ΔSUMO and SALL1DSIM mutants **(F,G)**. SALL1 does not colocalize with PML **(H)** nor with SC35 **(I)**. Green and magenta channels are shown independently in black and white. Nuclei were stained with DAPI (blue). White arrowheads indicate colocalization, green arrowheads indicate domains where mainly CBX4 **(A–D)** or SALL1 **(E–I)** proteins are present, magenta arrowheads indicate domains where mainly SUMO2 **(A)**, SUMO1 **(B,E–G)**, PML **(C,H)**, or SC35 **(D,I)** are present. Pictures were taken using a Leica DM IRE2 confocal microscope with a 63× objective, except for pictures in C that were taken using an AxioD Fluorescent microscope and objective 40×. Scale bars indicate 5 μm.

Supplementary Figure 3 SALL1 SUMOylation sites and SIMs are conserved throughout evolution. **(A)** SALL1 schematic representation. Ovals represent the zinc fingers (ZF) distributed along the protein. Blue rectangle represents the poly-glutamine (PQ) domain. In magenta, SUMO consensus sites mutated in SALL1ΔSUMO and, in blue, predicted SIMs mutated in SALL1ΔSIM. **(B)** SALL1 fused to HA tag was SUMOylated in the presence (black circles) of bioSUMO3, transiently transfected in HEK 293FT cells. Asterisks indicate the modified SALL1 (SUMO-SALL1) that is shifted if compared with the size of non-modified SALL1 (arrowhead). Anti-tubulin staining was used as a loading control. Molecular weight markers are shown to the right in KDa. SALL1ΔSUMO fused to HA tag is not SUMOylated in presence of bioSUMO3. In the input the expression of WT and SUMO mutant of SALL1 are shown. **(C)** In magenta, SUMO consensus sites in SALL1 that were mutated in SALL1ΔSUMO and, in blue, the predicted SIMs of SALL1, mutated in SALL1ΔSIM mutant. **(D)** Evolutionary conservation of the SUMOylation and SIM sites in SALL1 homologs in the indicated species. Asterisks indicate identical residues; colons and semicolons indicate conservative and semi-conservative changes, respectively.

Supplementary Figure 4 CBX4 and SALL1 localize to the nucleoplasm. Endogenous CBX4 **(A)** and endogenous SALL1 **(B)** shown in green localize to nuclear bodies in U2OS cells **(A′,B′)**. Increasing the intensity reveals the localization of both proteins in the nucleoplasm **(A″,B″)**. Single green channels are shown in black and white. Pictures were taken using a Leica DM IRE2 confocal microscope with a 63× objective.

Supplementary Figure 5 SALL1 SUMOylation is independent of CBX4. *In vitro* SUMOylation of SALL1 with SUMO1 or SUMO2/3 in the presence of growing quantities of CBX4 (in μl). Wheat germ was added as negative control. The vertical bar indicates the SUMOylated forms of SALL1, the empty arrowhead indicates the unmodified SALL1. Molecular weight markers are shown to the right in KDa.

Supplementary Figure 6 Variation of Polycomb bodies upon SALL1 expression. Representative composition of independent U2OS cells transfected with equal amounts of SALL1-YFP, SALL1ΔSUMO-YFP, or GFP-β-gal plasmids, stained for endogenous CBX4. Nuclei were labeled with DAPI (not shown). Pictures were taken using a Leica DM IRE2 confocal microscope with a 63× objective, using the same settings for all the conditions.
