# Supplementary figures and images for: SALL1 Modulates CBX4 Stability, Nuclear Bodies, and Regulation of Target Genes
Source: Front Cell Dev Biol. 2021 Sep 21;9:715868. doi: 10.3389/fcell.2021.715868 (PMC8490708; doi:10.3389/fcell.2021.715868)

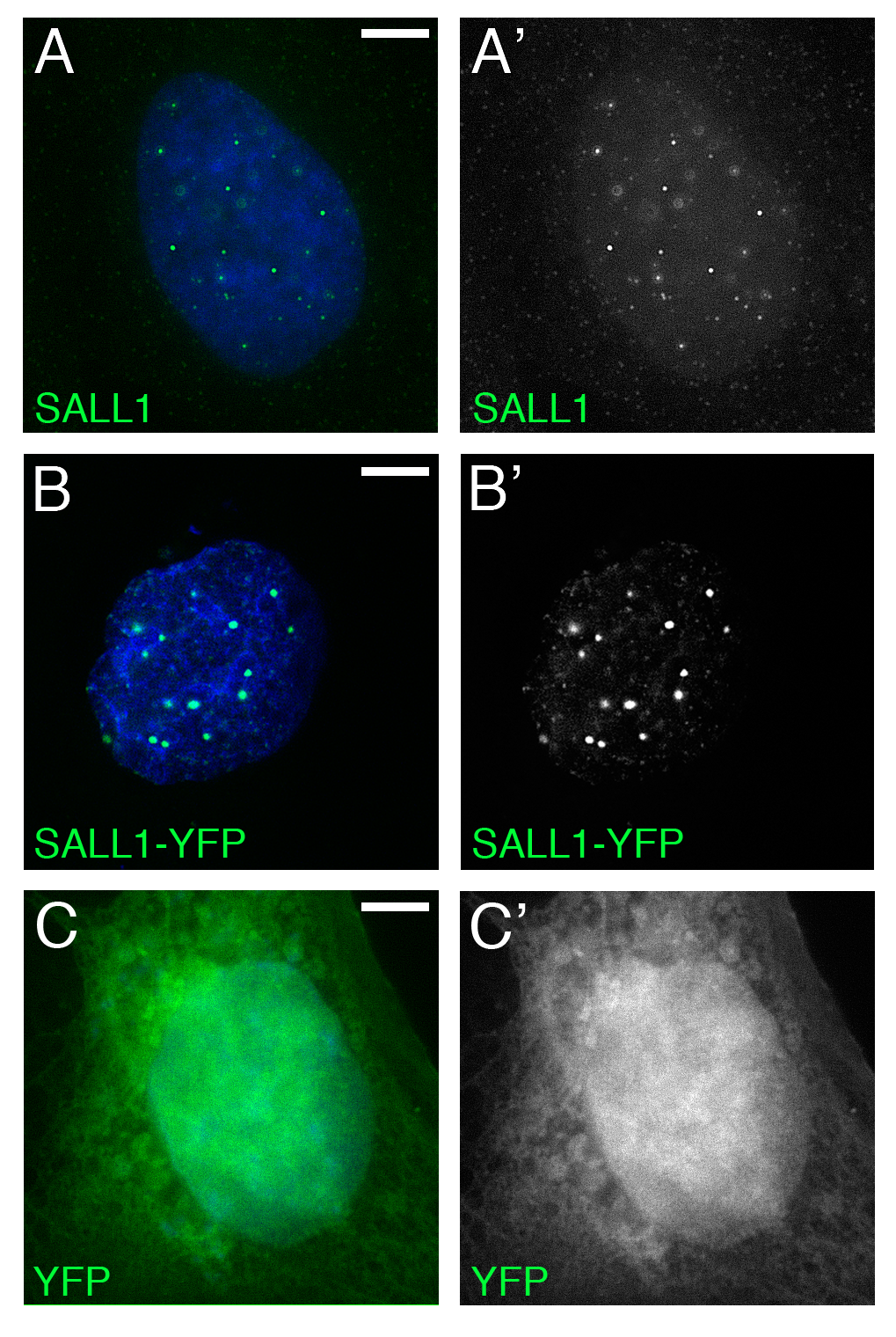

Supplement: Supplementary Figure 1 — SALL1 localizes to nuclear bodies. Endogenous SALL1 (A) and transiently expressed SALL1-YFP (B) localize to nuclear bodies in U2OS cells. In contrast, YFP alone, used as a control, shows a homogenous distribution in the nucleus and cytoplasm (C). Pictures were taken with an AxioD Fluorescent microscope using 100× objective. Scale bars indicate 5 μm. [file Image_1.TIF]

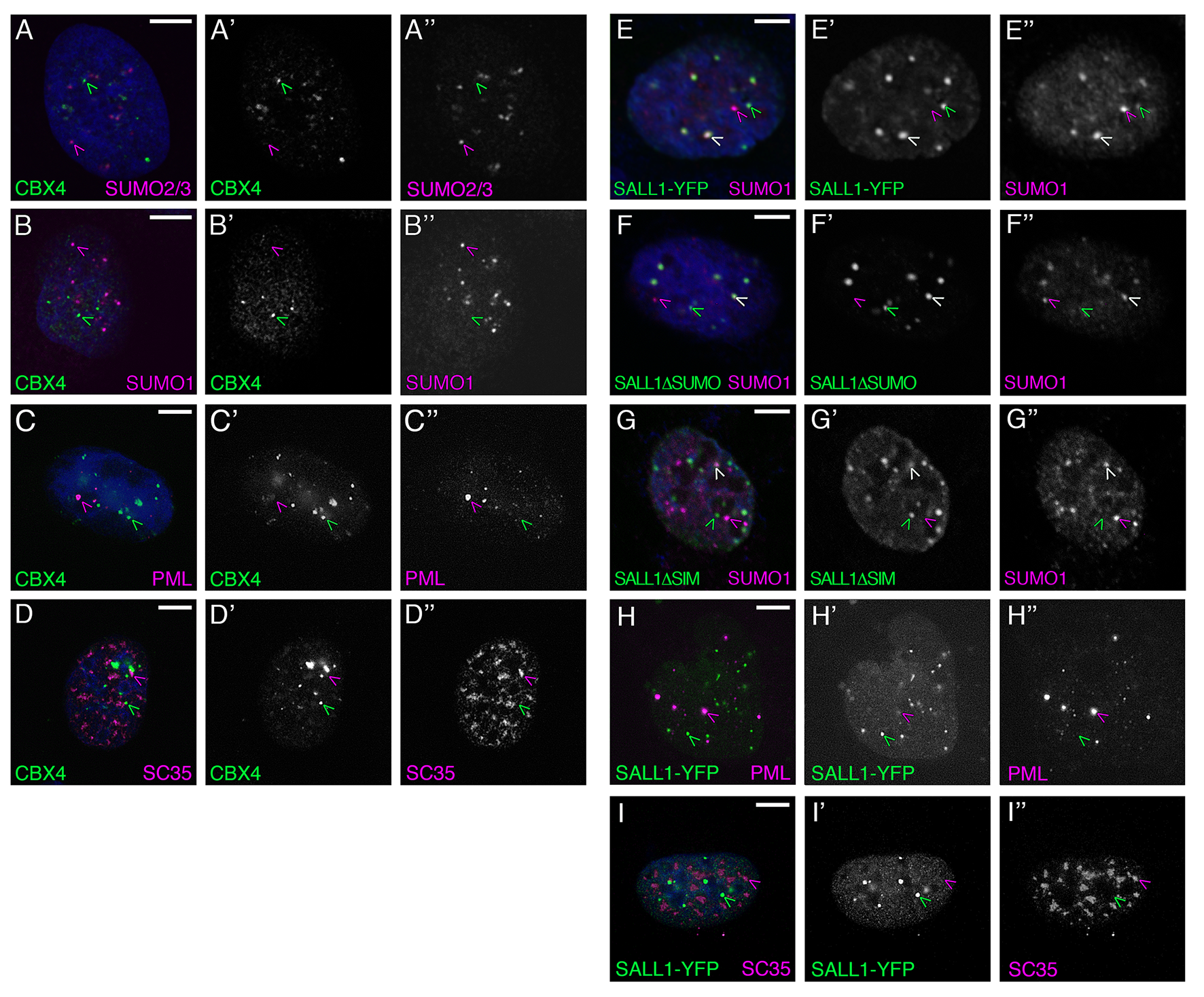

Supplement: Supplementary Figure 2 — Characterization of CBX4 and SALL1 nuclear bodies. (A–D) Endogenous CBX4 (green) does not colocalize with SUM2/3, SUMO1, nor PML bodies or with SC35 (magenta) in U2OS cells. (E–I) SALL1-YFP (green) partially colocalizes with endogenous SUMO1 (magenta) in U2OS cells (E). Similar results were obtained for the SALL1ΔSUMO and SALL1DSIM mutants (F,G). SALL1 does not colocalize with PML (H) nor with SC35 (I). Green and magenta channels are shown independently in black and white. Nuclei were stained with DAPI (blue). White arrowheads indicate colocalization, green arrowheads indicate domains where mainly CBX4 (A–D) or SALL1 (E–I) proteins are present, magenta arrowheads indicate domains where mainly SUMO2 (A), SUMO1 (B,E–G), PML (C,H), or SC35 (D,I) are present. Pictures were taken using a Leica DM IRE2 confocal microscope with a 63× objective, except for pictures in C that were taken using an AxioD Fluorescent microscope and objective 40×. Scale bars indicate 5 μm. [file Image_2.TIF]

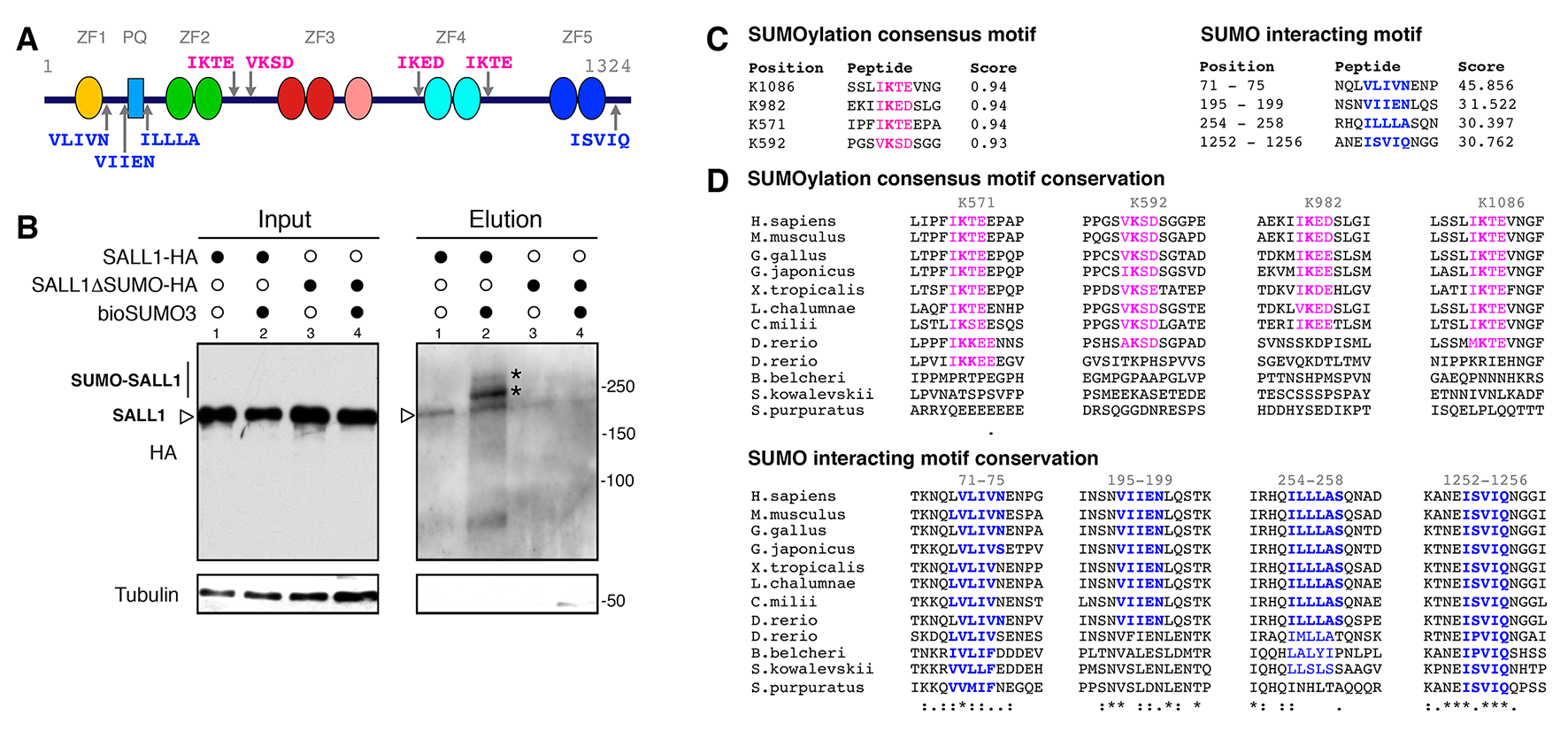

Supplement: Supplementary Figure 3 — SALL1 SUMOylation sites and SIMs are conserved throughout evolution. (A) SALL1 schematic representation. Ovals represent the zinc fingers (ZF) distributed along the protein. Blue rectangle represents the poly-glutamine (PQ) domain. In magenta, SUMO consensus sites mutated in SALL1ΔSUMO and, in blue, predicted SIMs mutated in SALL1ΔSIM. (B) SALL1 fused to HA tag was SUMOylated in the presence (black circles) of bioSUMO3, transiently transfected in HEK 293FT cells. Asterisks indicate the modified SALL1 (SUMO-SALL1) that is shifted if compared with the size of non-modified SALL1 (arrowhead). Anti-tubulin staining was used as a loading control. Molecular weight markers are shown to the right in KDa. SALL1ΔSUMO fused to HA tag is not SUMOylated in presence of bioSUMO3. In the input the expression of WT and SUMO mutant of SALL1 are shown. (C) In magenta, SUMO consensus sites in SALL1 that were mutated in SALL1ΔSUMO and, in blue, the predicted SIMs of SALL1, mutated in SALL1ΔSIM mutant. (D) Evolutionary conservation of the SUMOylation and SIM sites in SALL1 homologs in the indicated species. Asterisks indicate identical residues; colons and semicolons indicate conservative and semi-conservative changes, respectively. [file Image_3.TIF]

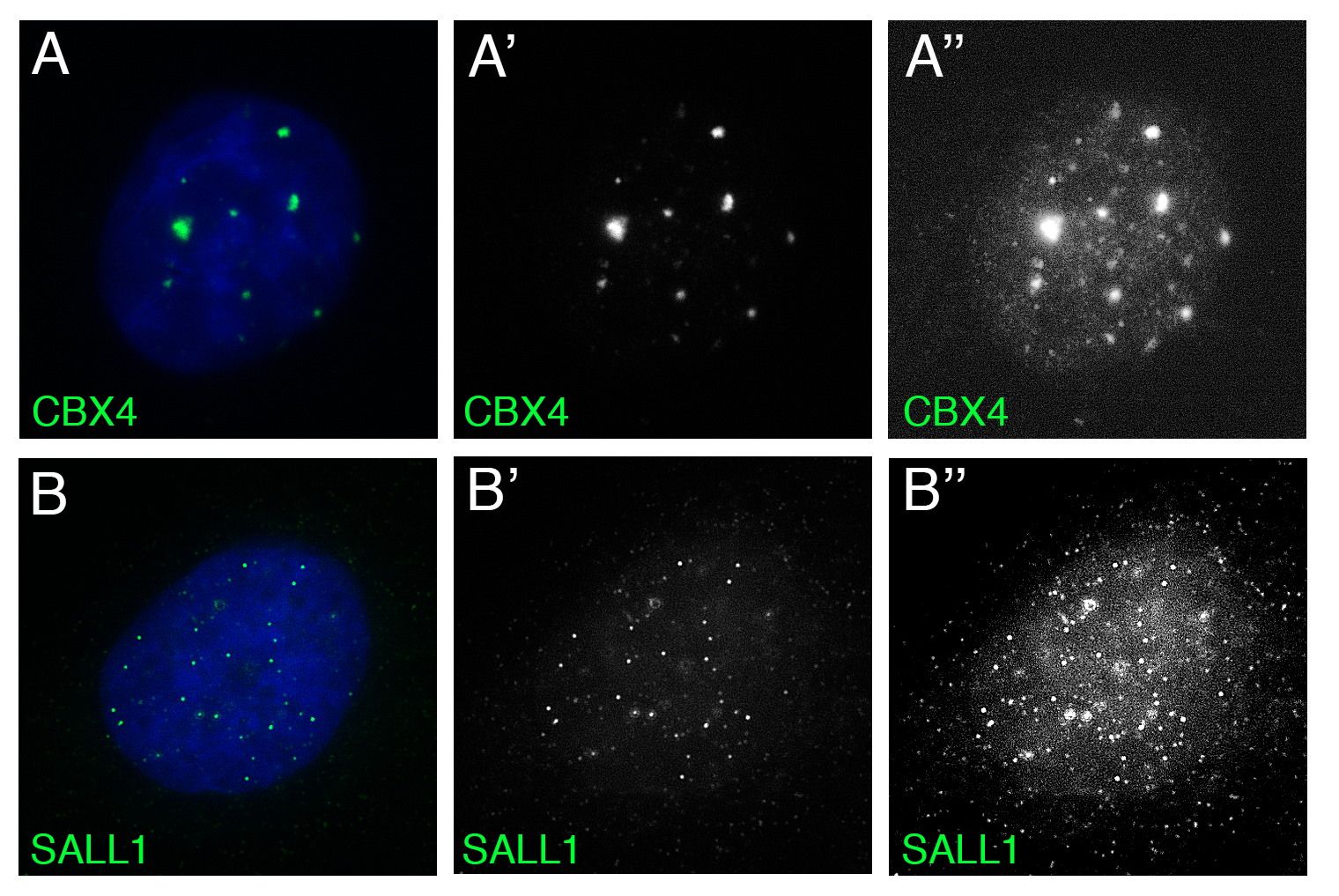

Supplement: Supplementary Figure 4 — CBX4 and SALL1 localize to the nucleoplasm. Endogenous CBX4 (A) and endogenous SALL1 (B) shown in green localize to nuclear bodies in U2OS cells (A′,B′). Increasing the intensity reveals the localization of both proteins in the nucleoplasm (A″,B″). Single green channels are shown in black and white. Pictures were taken using a Leica DM IRE2 confocal microscope with a 63× objective. [file Image_4.TIF]

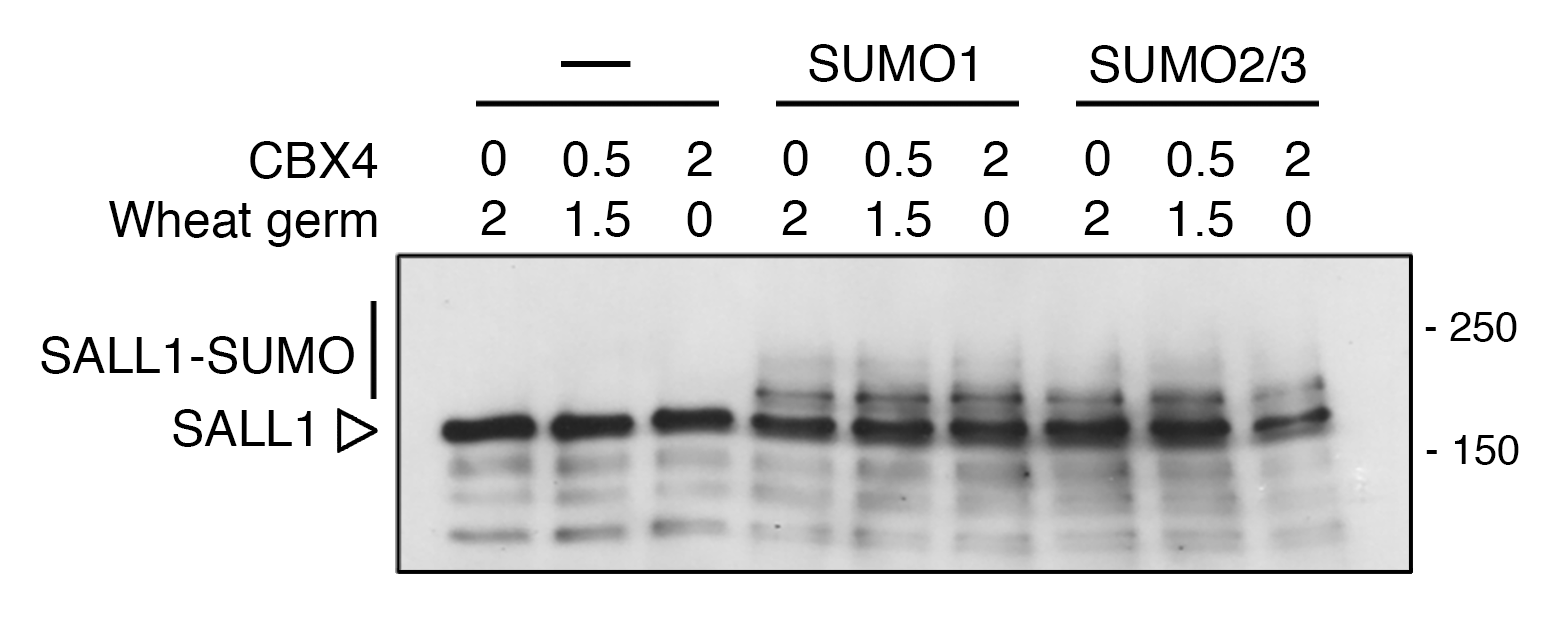

Supplement: Supplementary Figure 5 — SALL1 SUMOylation is independent of CBX4. In vitro SUMOylation of SALL1 with SUMO1 or SUMO2/3 in the presence of growing quantities of CBX4 (in μl). Wheat germ was added as negative control. The vertical bar indicates the SUMOylated forms of SALL1, the empty arrowhead indicates the unmodified SALL1. Molecular weight markers are shown to the right in KDa. [file Image_5.TIF]

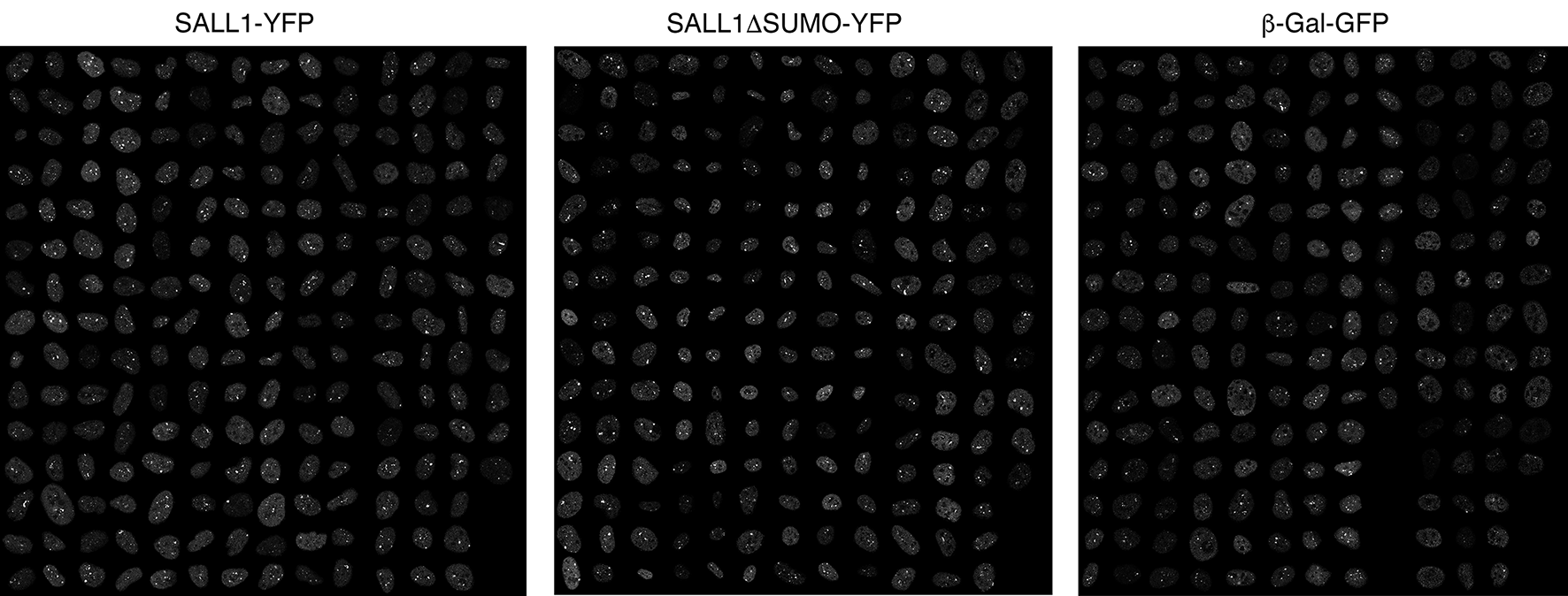

Supplement: Supplementary Figure 6 — Variation of Polycomb bodies upon SALL1 expression. Representative composition of independent U2OS cells transfected with equal amounts of SALL1-YFP, SALL1ΔSUMO-YFP, or GFP-β-gal plasmids, stained for endogenous CBX4. Nuclei were labeled with DAPI (not shown). Pictures were taken using a Leica DM IRE2 confocal microscope with a 63× objective, using the same settings for all the conditions. [file Image_6.TIF]
